# Supplementary material for: Characterization of LC-MS based urine metabolomics in healthy children and adults
Source: PeerJ. 2022 Jun 22;10:e13545. doi: 10.7717/peerj.13545 (PMC9233480; doi:10.7717/peerj.13545)
Supplement: Supplemental Information 1 — Data processing using Progenesis QI [file peerj-10-13545-s001.docx]

Characterization of LC-MS based urine metabolomics in healthy children and adults

**Data processing using Progenesis QI**

The detailed workflow for data processing facilitated by Progenesis QI is involved “create a new experiment”, “import data”, “review alignment”, “experiment design setup”, “peak picking”, “reviewed convolution”, and “identify compounds” in sequence. In general, the whole process ran automatically using optimized parameter settings. (1) In the stage of create a new experiment, adduct ion was carefully selected as it would influence the number of characterized compounds and also the identification accuracy. Based on the ionization behaviors of reference standards, the adduct ion forms, comprising [M + H]+, [M + Na]+, [M + K], [M + NH4]+, [2M + H]+, [2M + Na]+,[2M + NH4]+, [M + H – H2O]+ and [M + H – 2H2O]+ , were selected. (2) The MS data acquired by LC-MS for all the URINE samples were imported into the Progenesis QI software, generating a 2D ion intensity map with the retention time and m/z information as the ordinate and abscissa, respectively. (3) Peak alignment was carried out in automatic manner taking a QC run as the reference, the score values for all the samples were greater than 90 %. (4) For peak picking, the thresholds of chromatographic peak absolute intensity, and retention time limits can be set to achieve the maximum real ion signals with noise excluded. In the present study, absolute intensity and retention time limit were set at 1000 and default. (5) Further compound identification was performed by searching the HMDB database (2018 version). The MS1 mass tolerance was set as 10 ppm and the MS/MS mass tolerance was set as 20 ppm.
