# Supplementary material for: Characterization of LC-MS based urine metabolomics in healthy children and adults
Source: PeerJ. 2022 Jun 22;10:e13545. doi: 10.7717/peerj.13545 (PMC9233480; doi:10.7717/peerj.13545)

Characterization of LC-MS based urine metabolomics in healthy children and adults

**FigS1** a. Score plot of all samples. Good cluster of QC samples indicated good stability of the analytical platform. Separation of adults and children samples resulted from biological or sampling center differences. b. PCA and OPLS-DA score plot of urine metabolomics between male and females in children. c. PCA and OPLS-DA score plot of urine metabolomics between male and females in adults. 100 permutation test was used to validate the OPLS-DA model. The permutation plots were given in “b” and “c”.


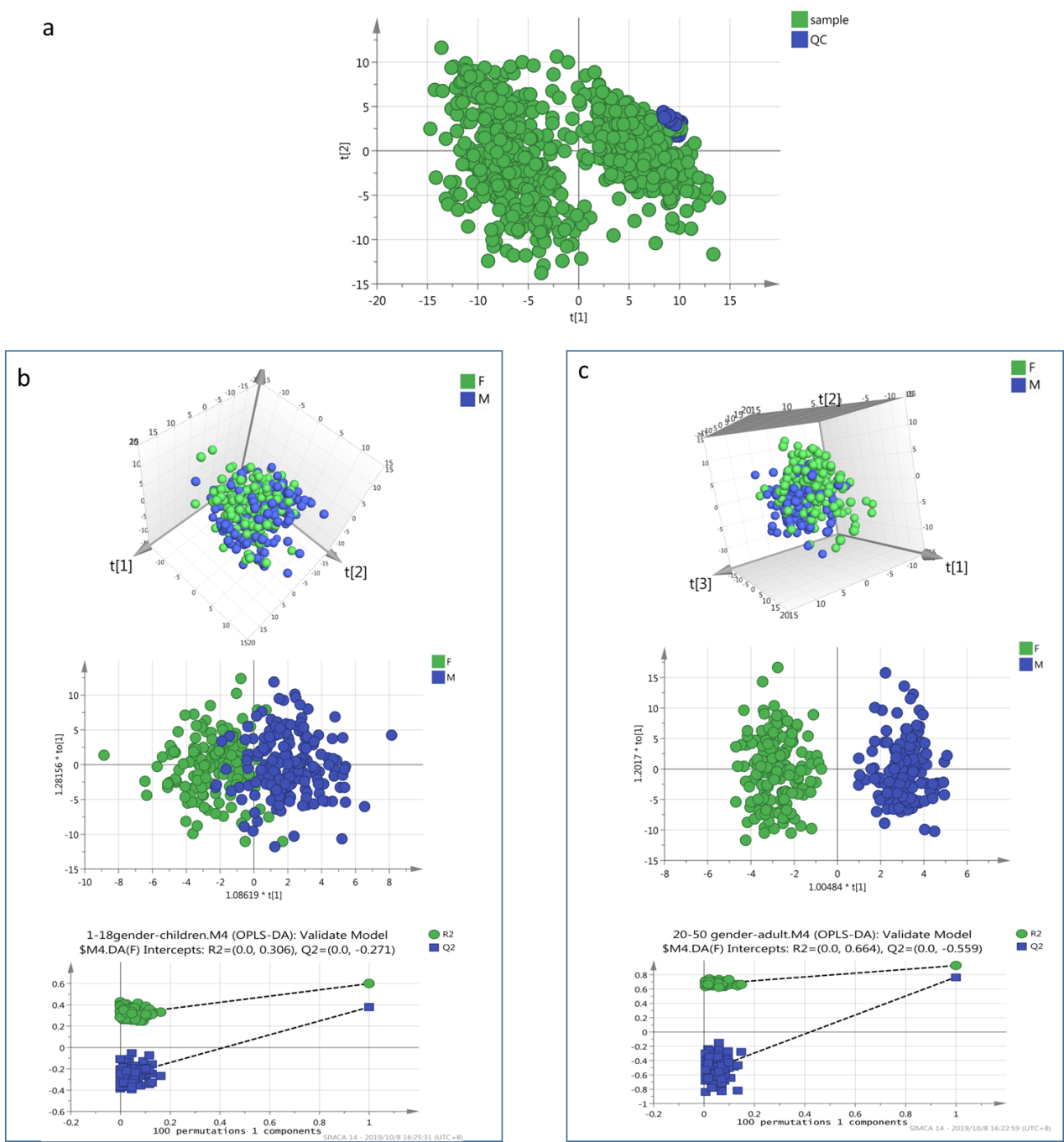


**Fig S2** Separation of urine metabolites with different age stages in children and adults. A. PCA score plot of urine metabolomics in different age group for children-boys, children-girls, adults-males and adults-females. b. Permutation test plots of PLS-DA model based on different age group for children-boys, children-girls, adults-males and adults-females. c. PLS-DA score plot of urine metabolomics of different age in males. d. PLS-DA score plot of urine metabolomics of different age in females. e. Change trend of the first and second components of PLS-DA in adults. The first component explain the most variance of the model, thus contributing most to class separation.


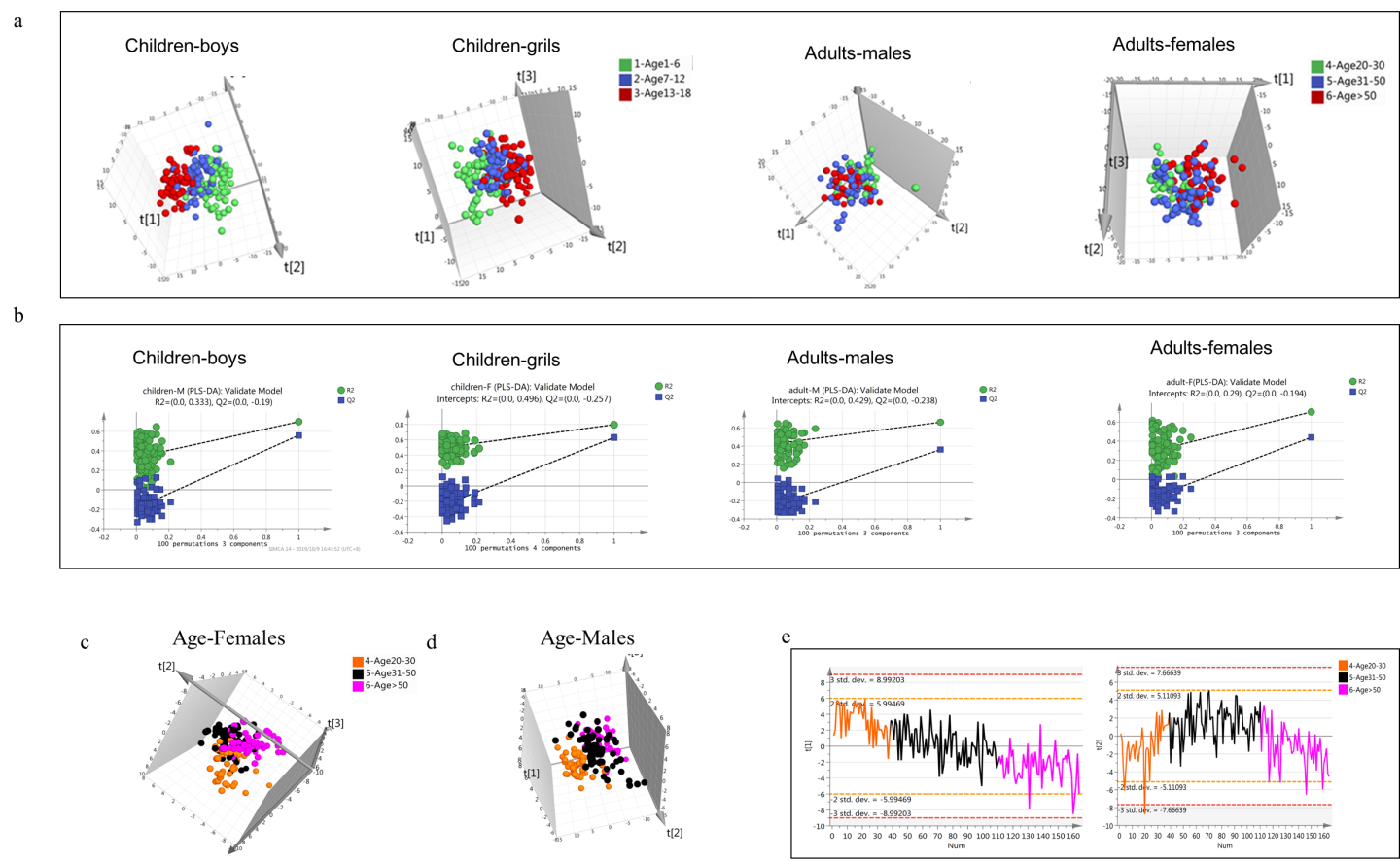


Fig S3 Metabolites annotation using database and standards spectra or fragments annotation

**Aspartyl-Valine(Score:53.2; Fragmentation score:75.9)**

**
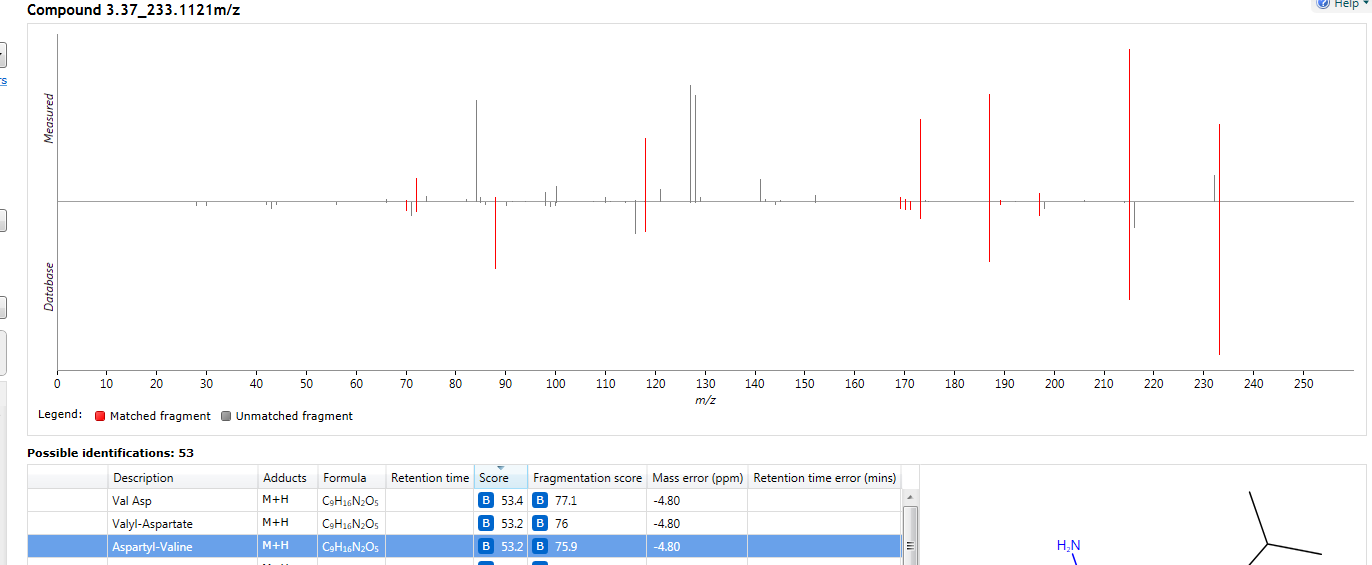
**

**1,9-Dimethyluric acid(Score:42.7; Fragmentation score:47.8)**

**
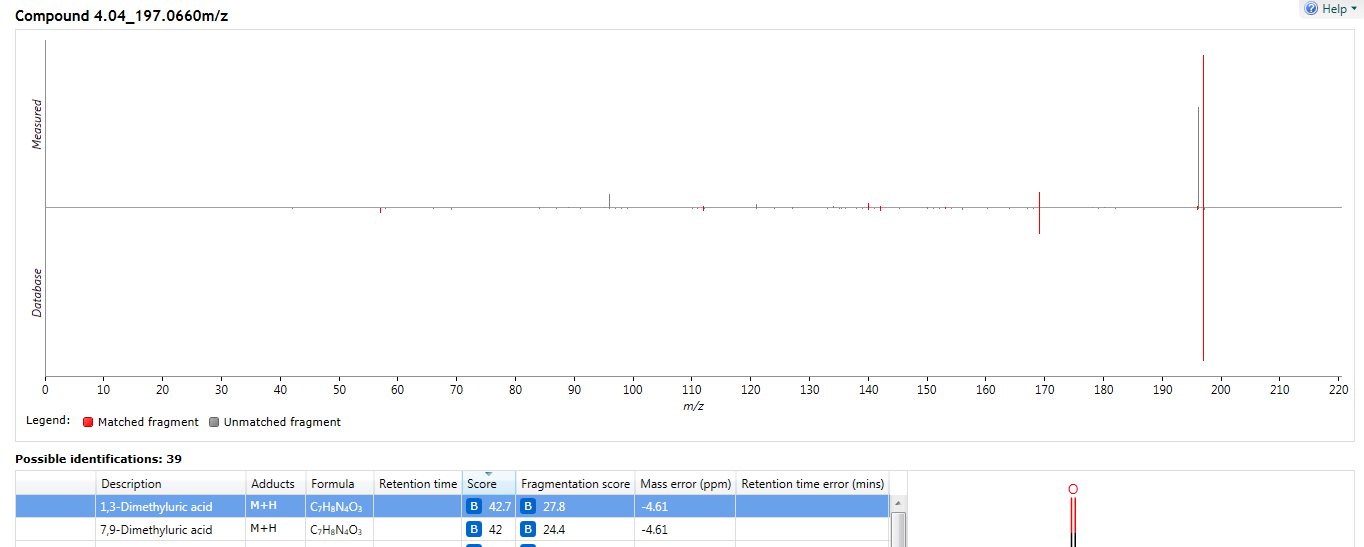
**

**5-Methylcytidine (Score:50.4; Fragmentation score:68.9)**

**
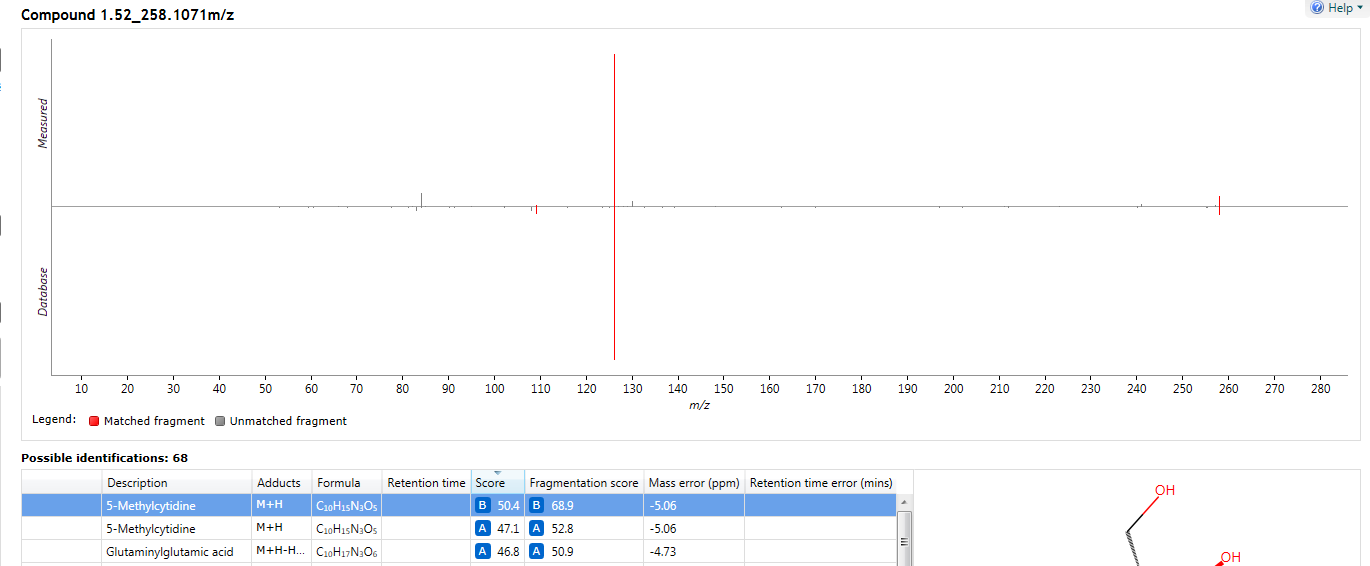
**

**Theobromine (Score:46.4; Fragmentation score:44.6)**

**
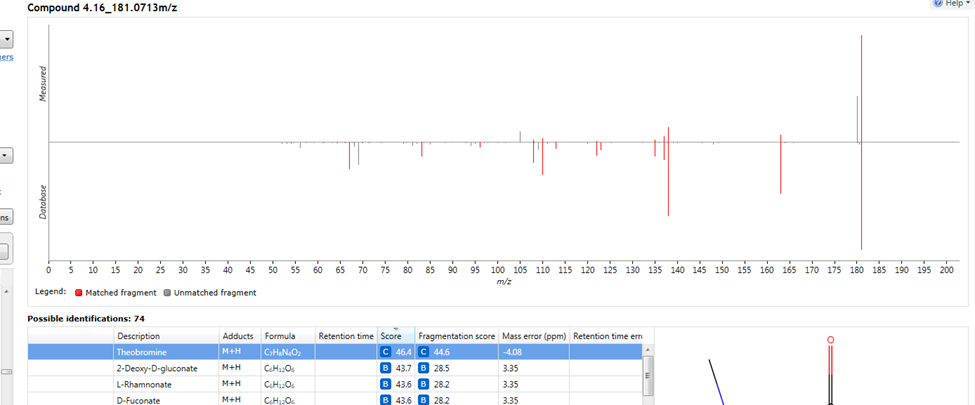
**

**3-Methoxytyrosine(Score:55.4; Fragmentation score:78.9)**

**
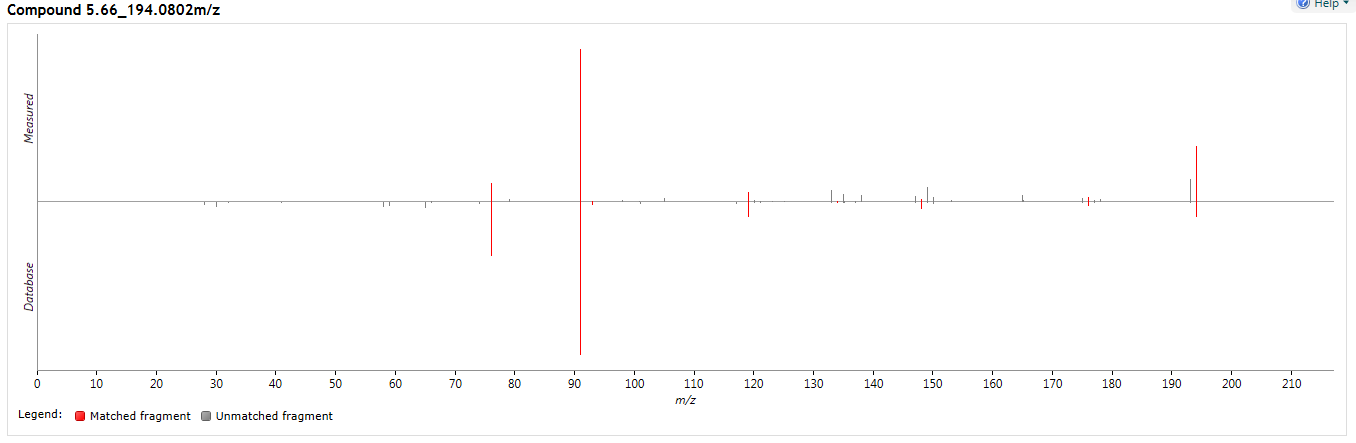
**

**5-Hydroxyindoleacetic acid (Score:50.1; Fragmentation score:62.2)**

**
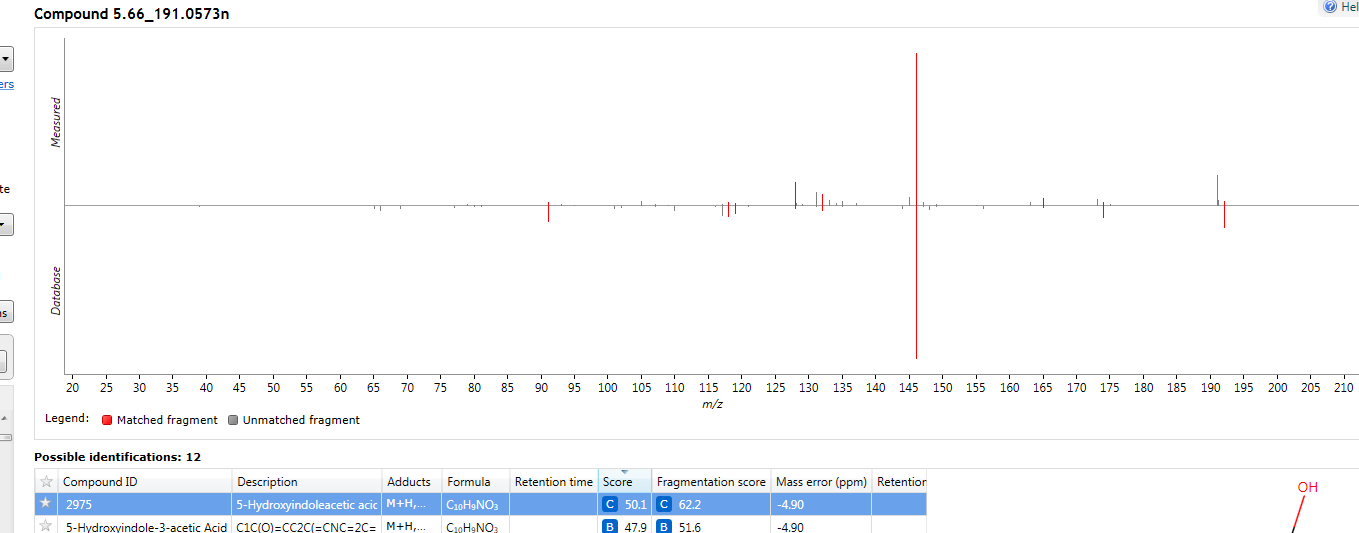
**

**9-Methyluric acid (Score:46.6; Fragmentation score:46.2)**

**
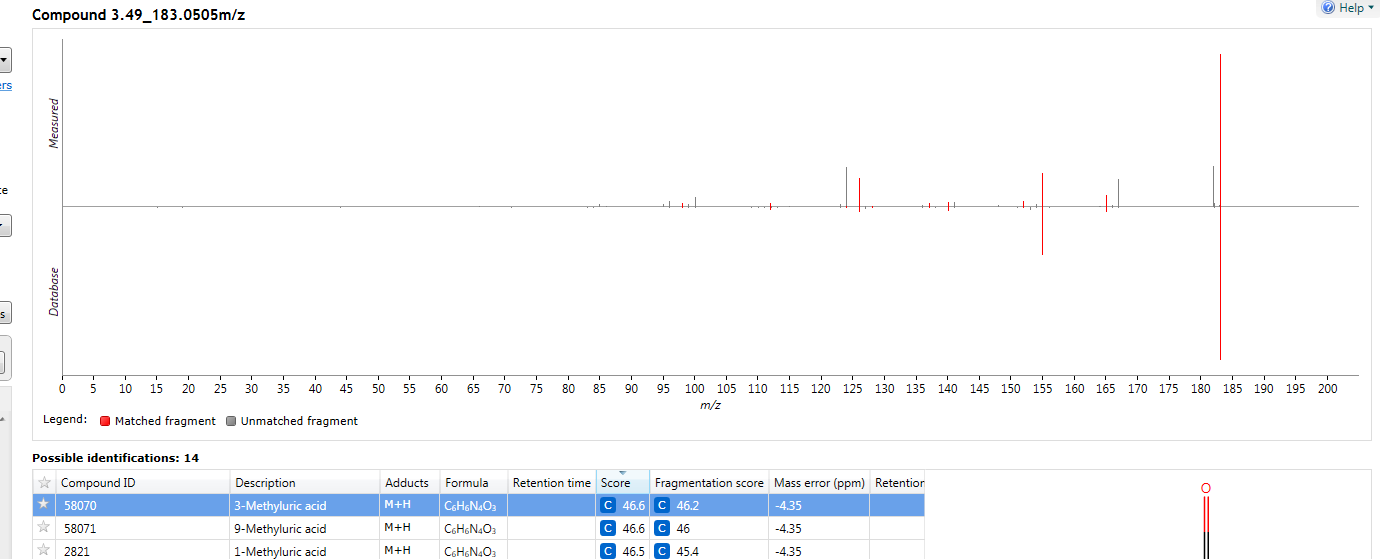
**

**Deoxyinosine (Score:49.1; Fragmentation score:62.6)**

**
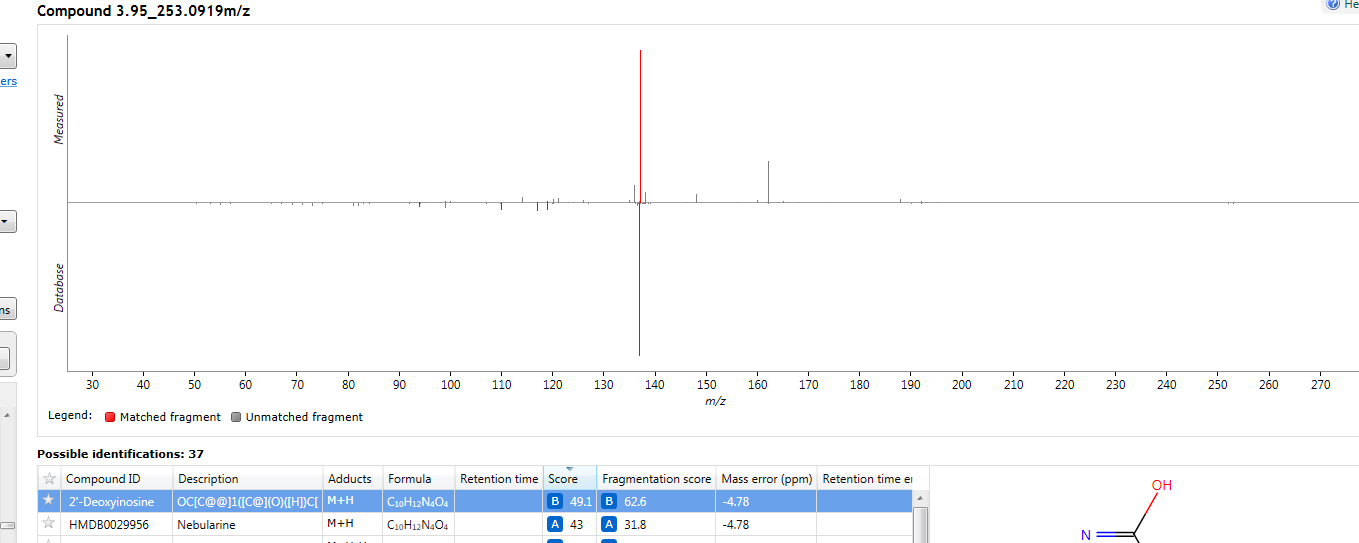
**

**Riboflavin (Score:52.2; Fragmentation score:74.5)**

**
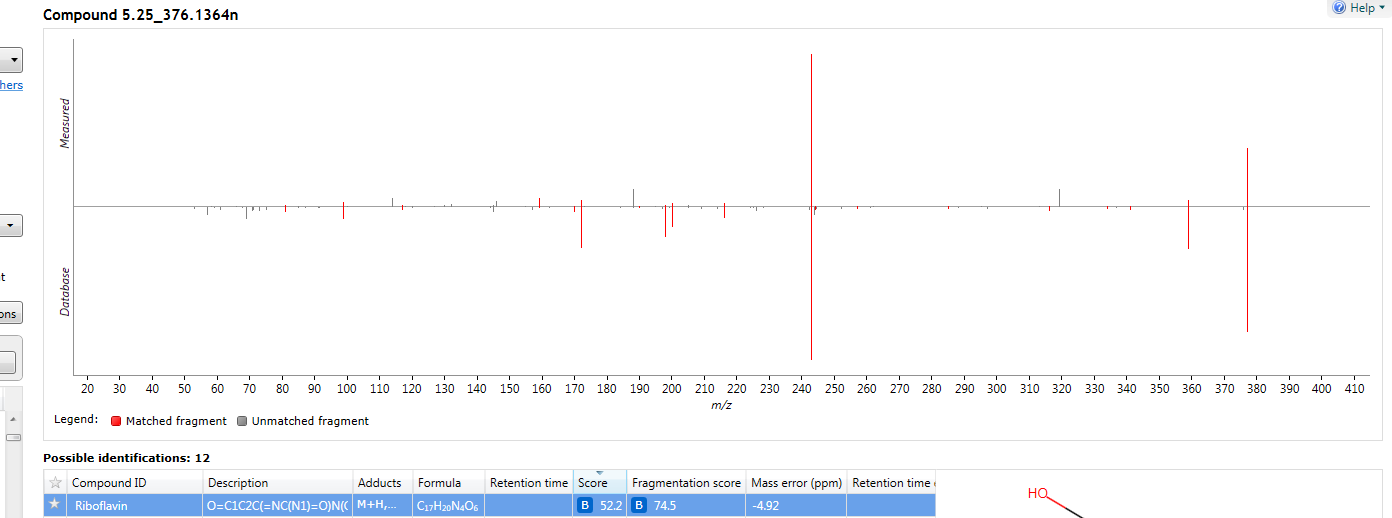
**

**Cinnamoylglycine(Score:54.2; Fragmentation score:82.4)**

**
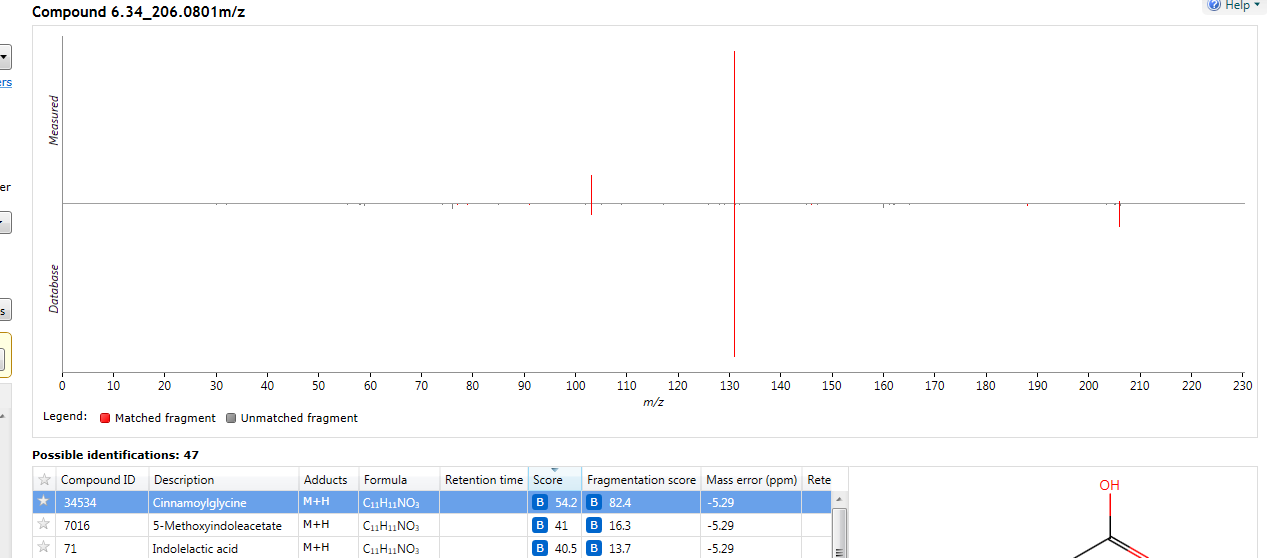
**

**Tyrosyl-Hydroxyproline (Score:49.4; Fragmentation score:60.1)**

**
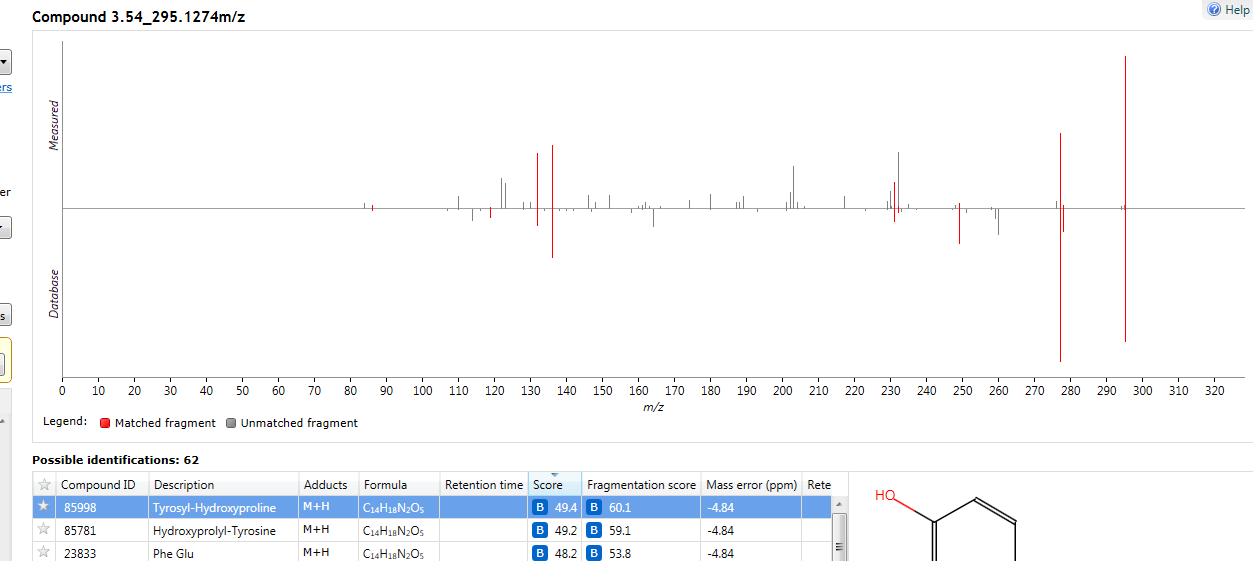
**

**5-Acetylamino-6-amino-3-methyluracil (Score:54.5; Fragmentation score:65.2)**

**
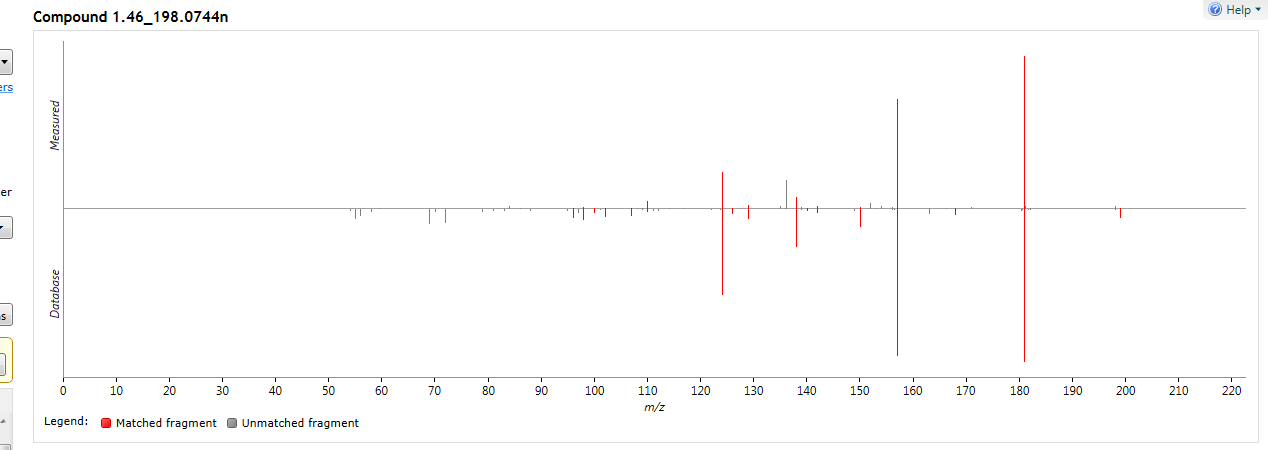
**

**Pantothenic acid (Score:65.8; Fragmentation score:89.4)**

**
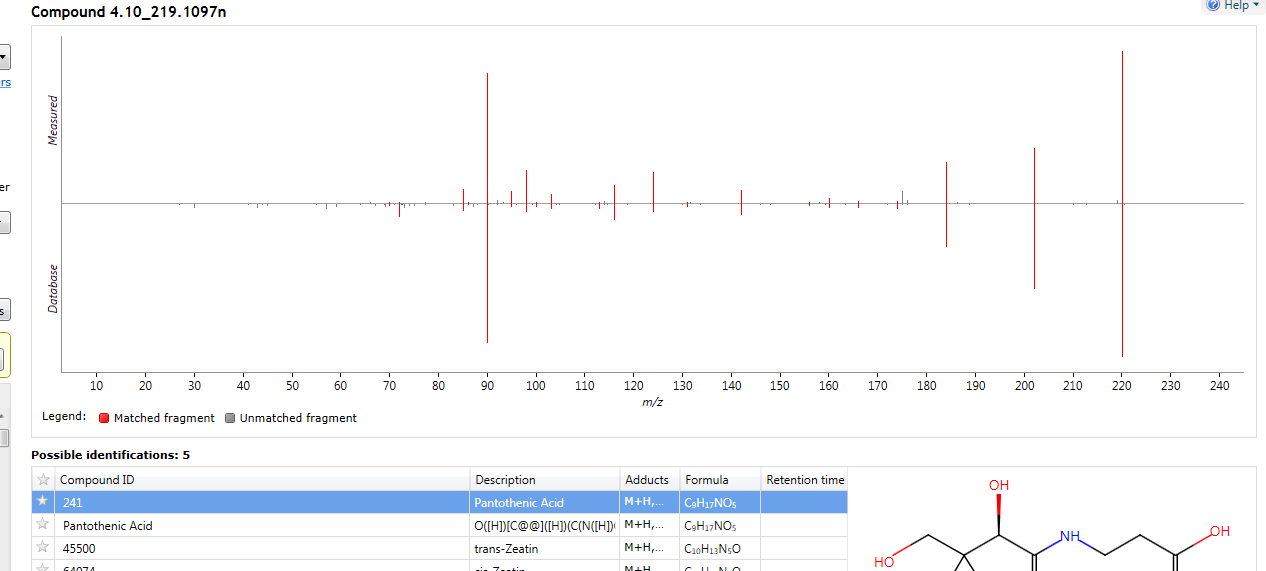
**

**Uric acid (Score:65.4; Fragmentation score:70.5)**

**
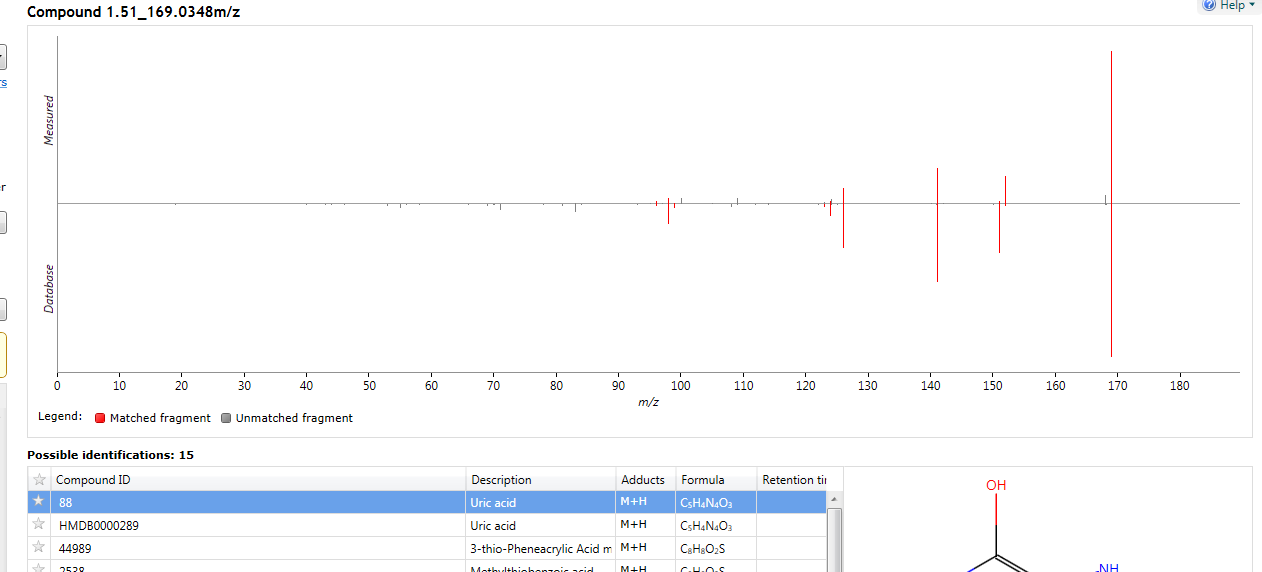
**

**2-Furoylglycine (Score:46.7; Fragmentation score:44)**

**
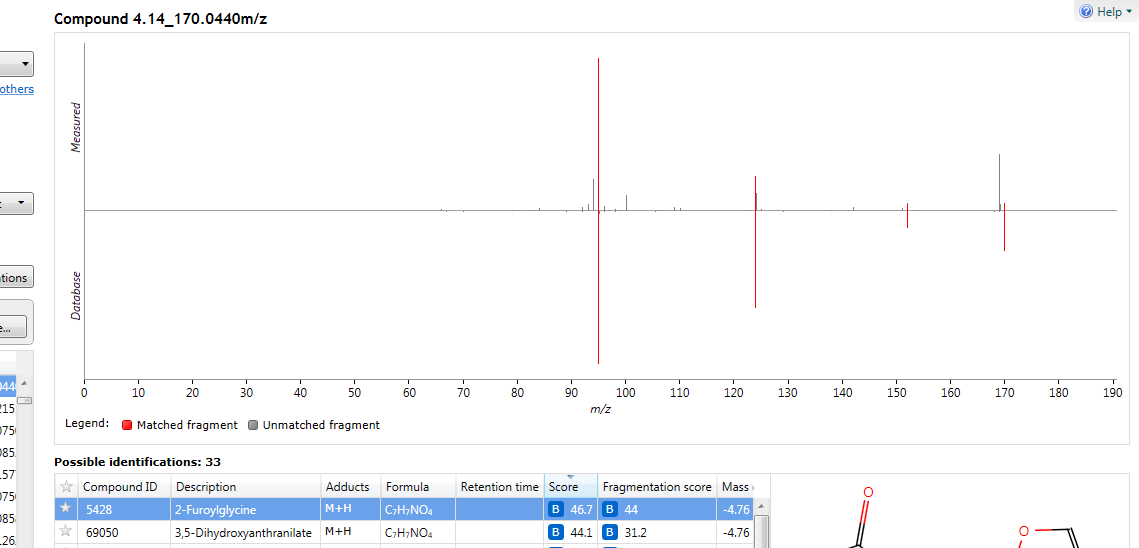
**

**S-Adenosylhomocysteine(Score:51.4; Fragmentation score:89.1)**

**
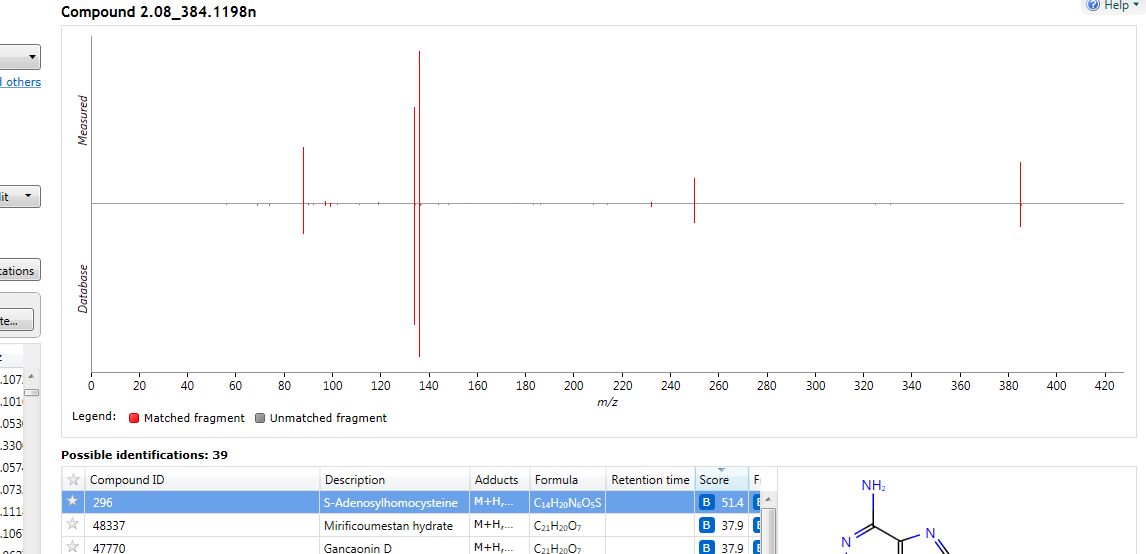
**

**N-Acetylcadaverine (Score:46.8; Fragmentation score:50.3)**

**
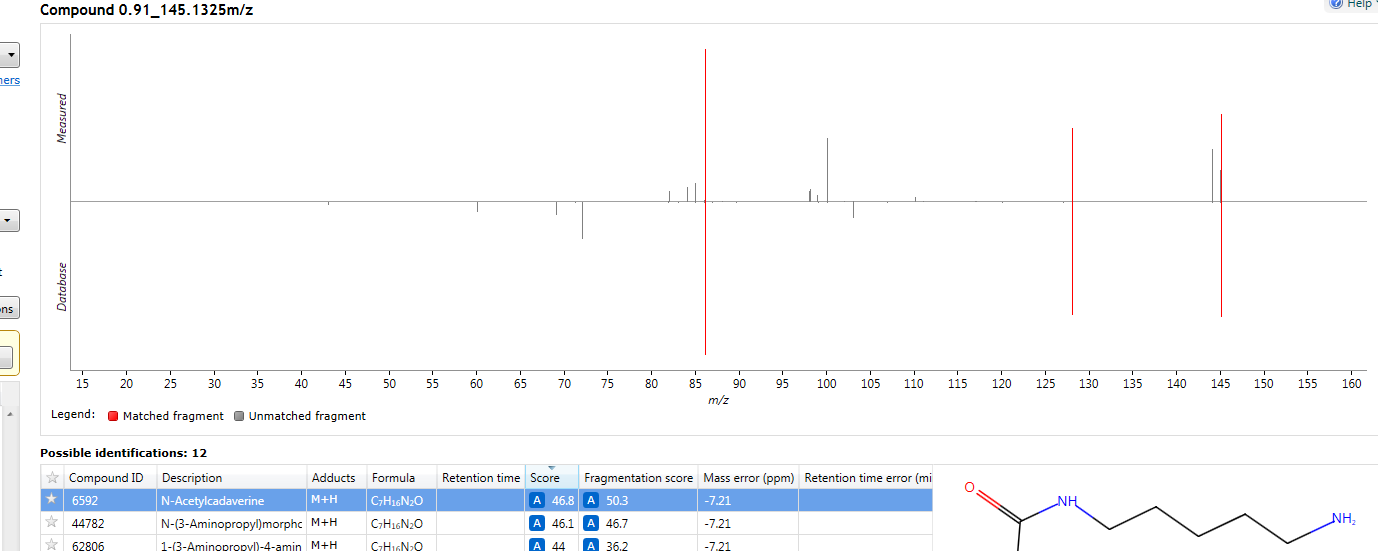
**

**Glutamylphenylalanine (Score:49.2; Fragmentation score:58.1)**

**
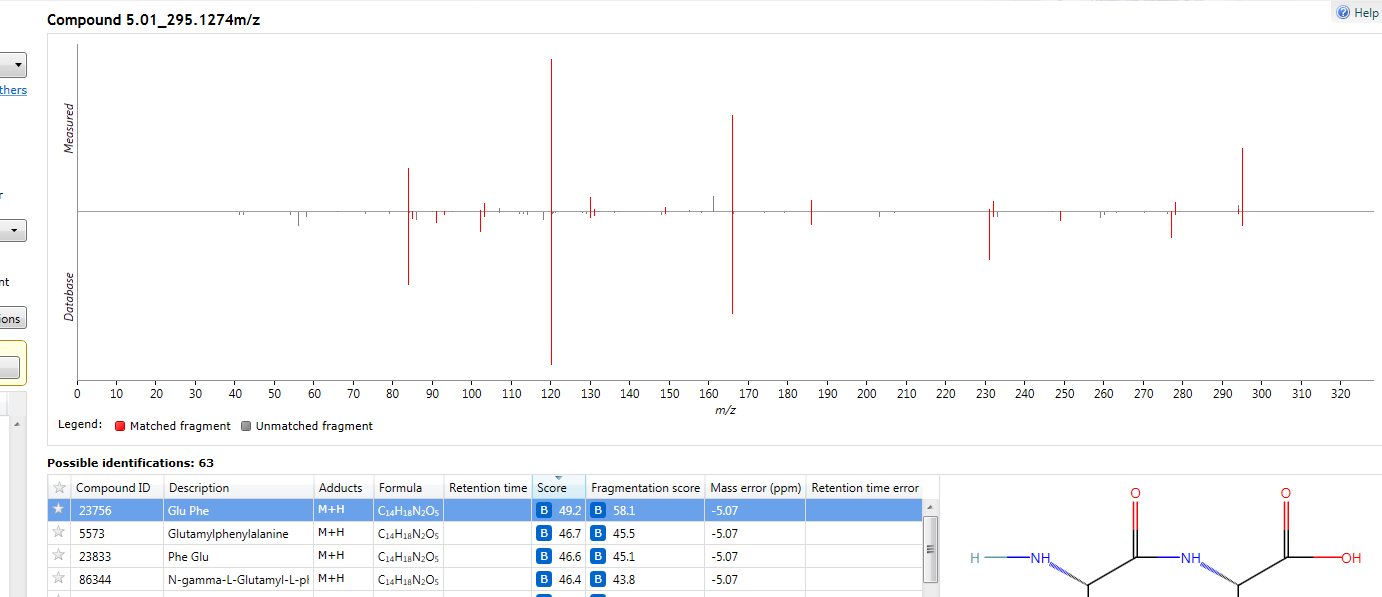
**

**L-Glutamine (Score:48.7; Fragmentation score:62.3)**

**
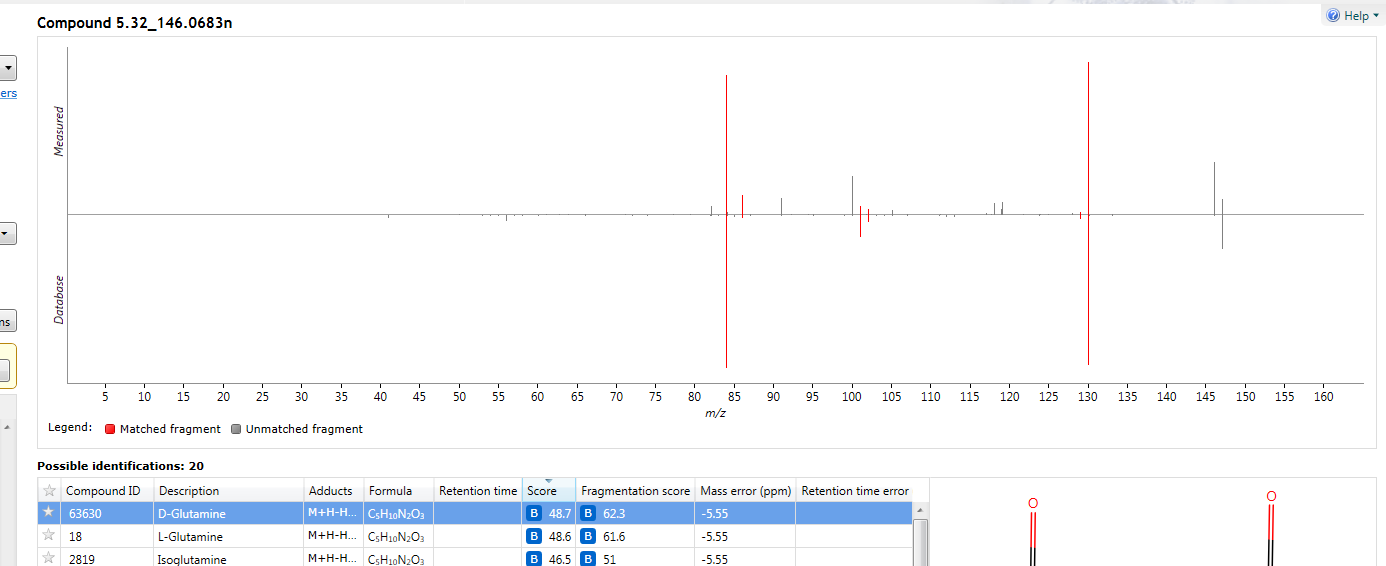
**

**Urocanic acid(Score:47; Fragmentation score:46.5)**

**
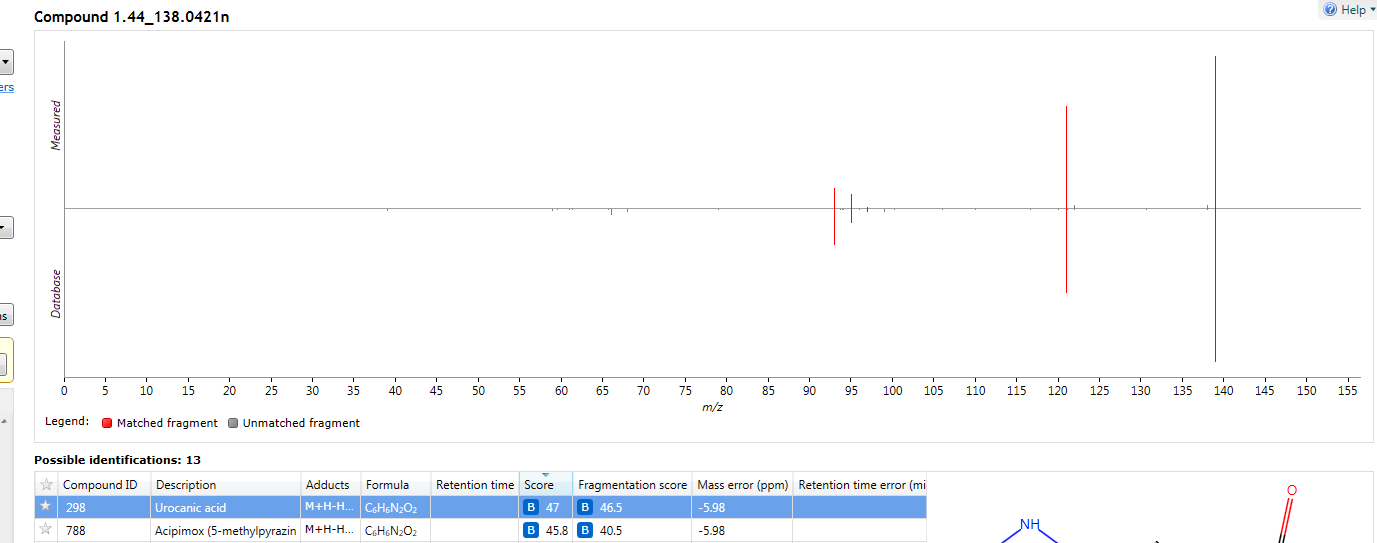
**

**L-Octanoylcarnitine(Score:52.2; Fragmentation score:73.1)**

**
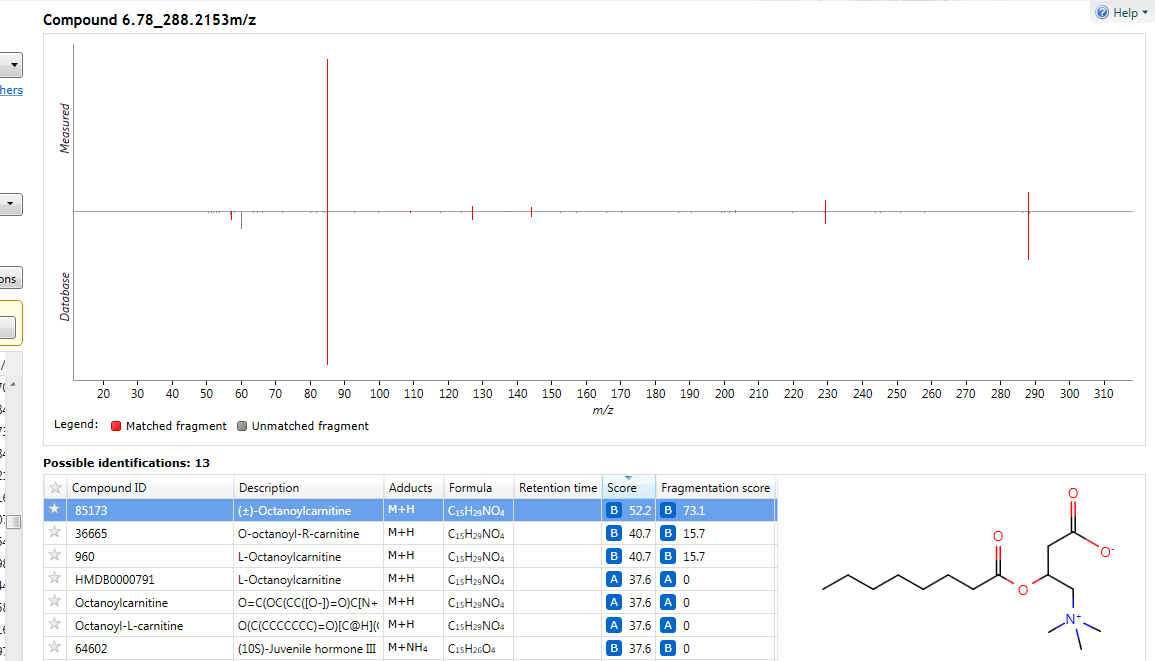
**

**4-Pyridoxic acid (Score:50.1; Fragmentation score:62.9)**

**
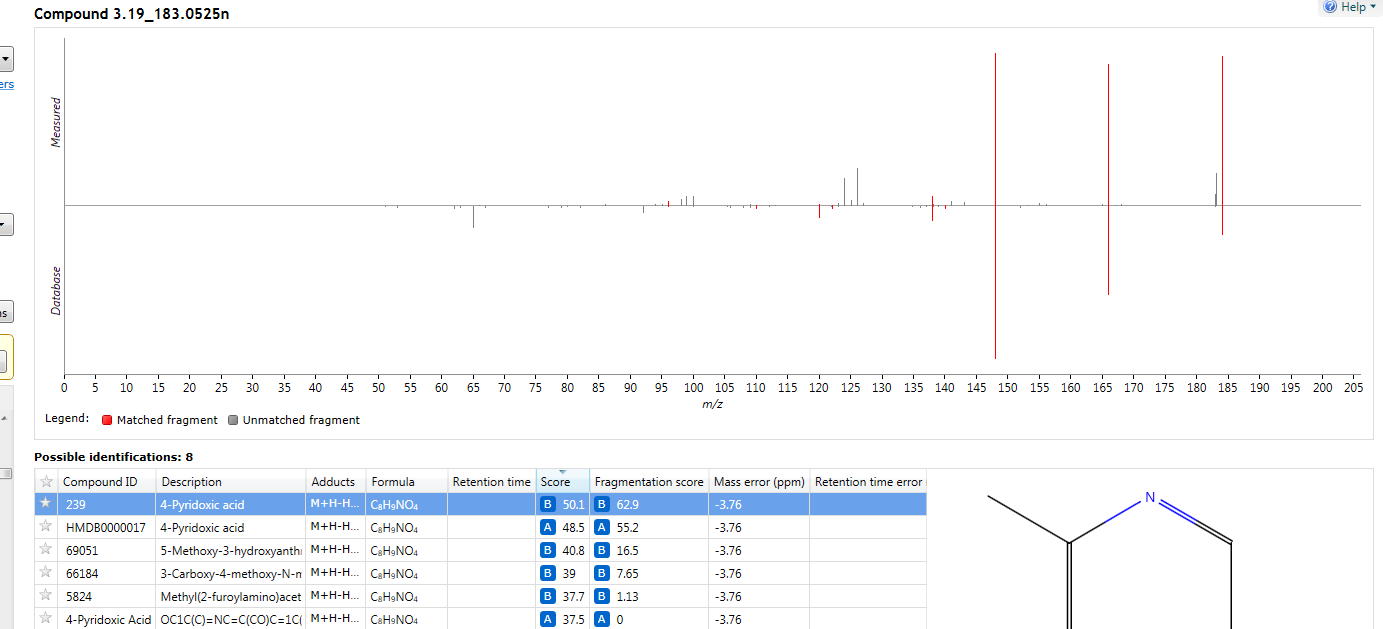
**

**Succinyladenosine (Score:49.8; Fragmentation score:58.6)**

**
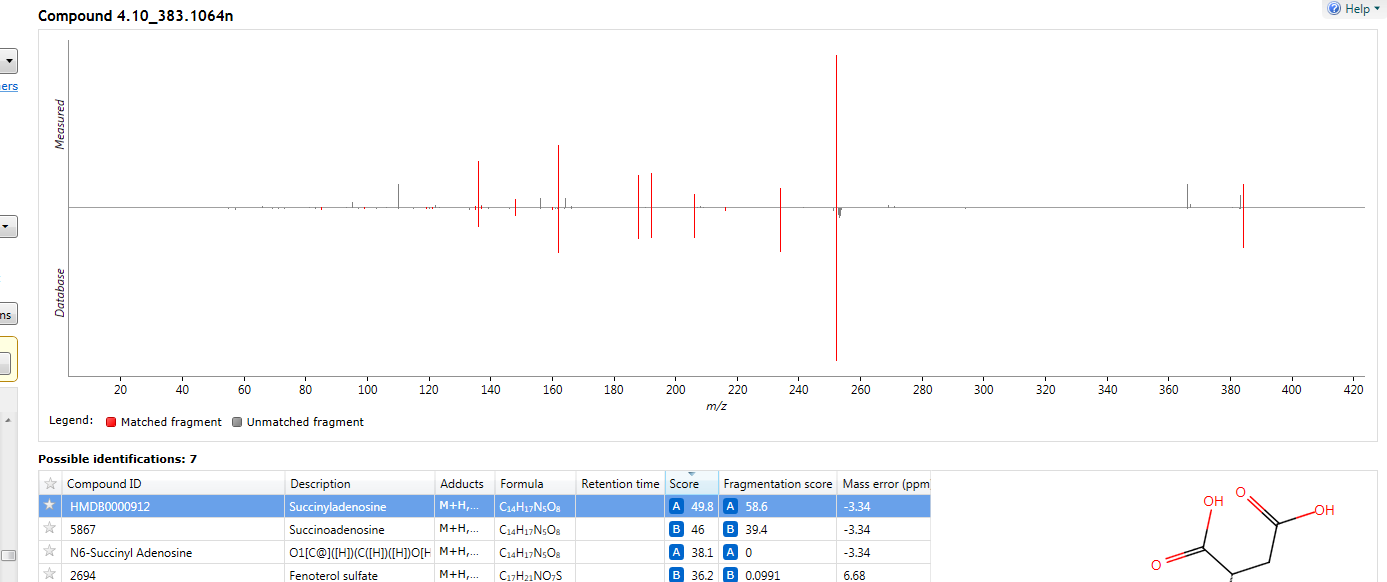
**

**Tetrahydrocortisone (Score:47; Fragmentation score:47.5)**

**
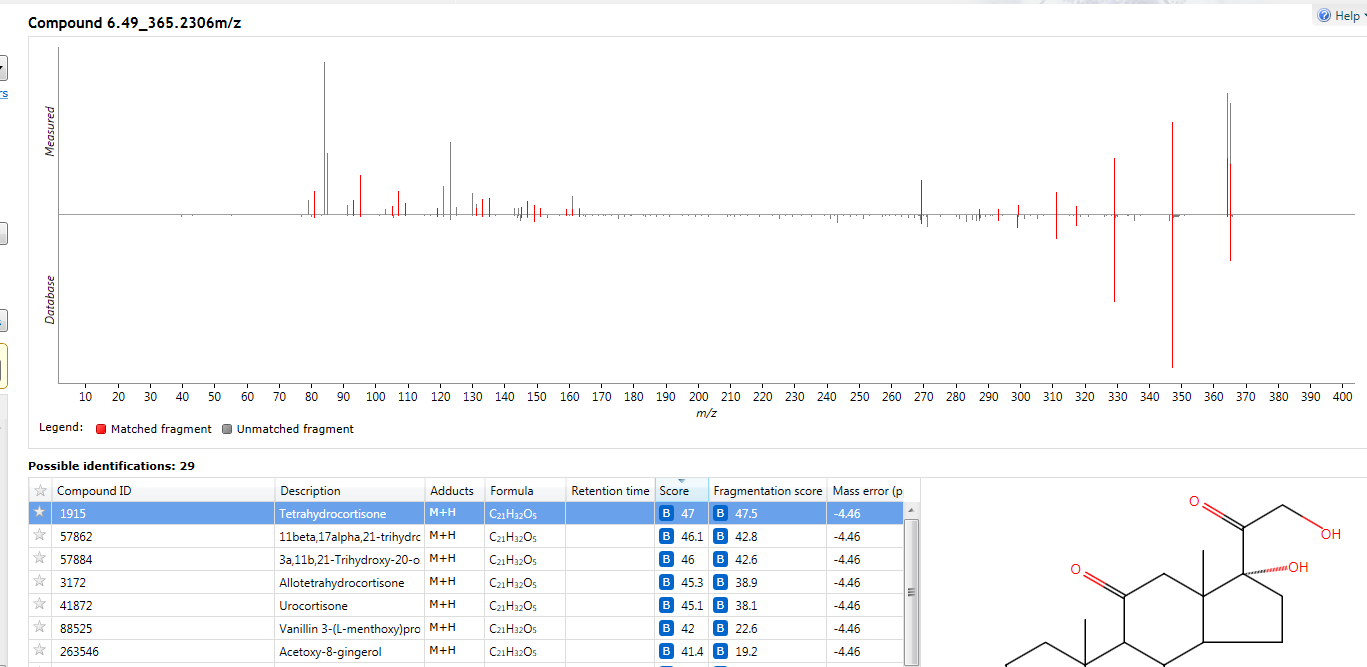
**

**Methyldopa (Score:50.5; Fragmentation score:67.6)**

**
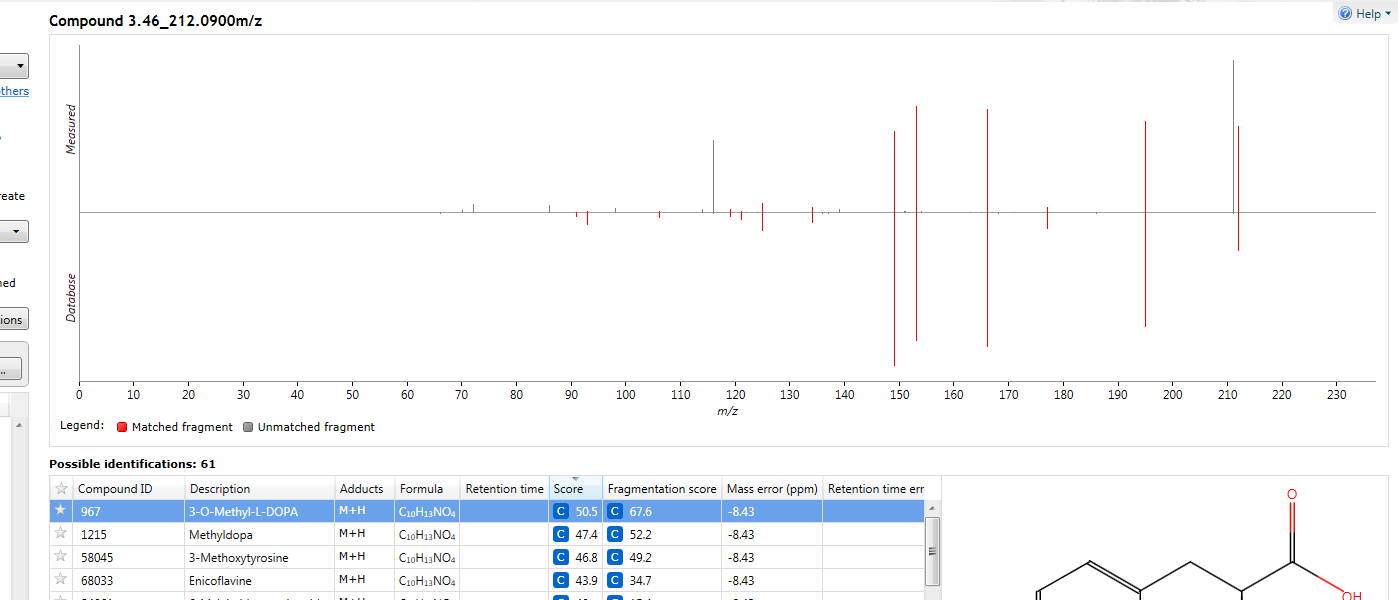
**

**L-beta-aspartyl-L-leucine/** **gamma-Glutamylvaline (Score:53.2; Fragmentation score:77)**

**
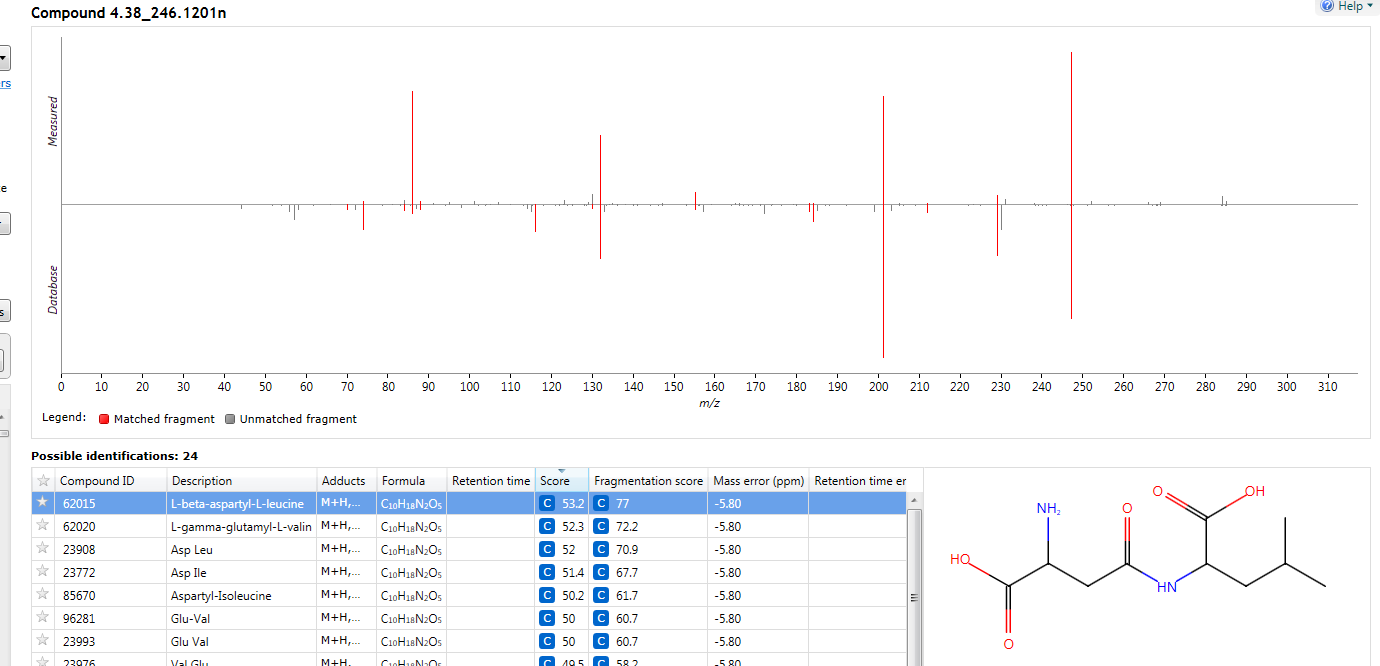
**

**2-Methylguanosine (Score:51.4; Fragmentation score:70.4)**

**
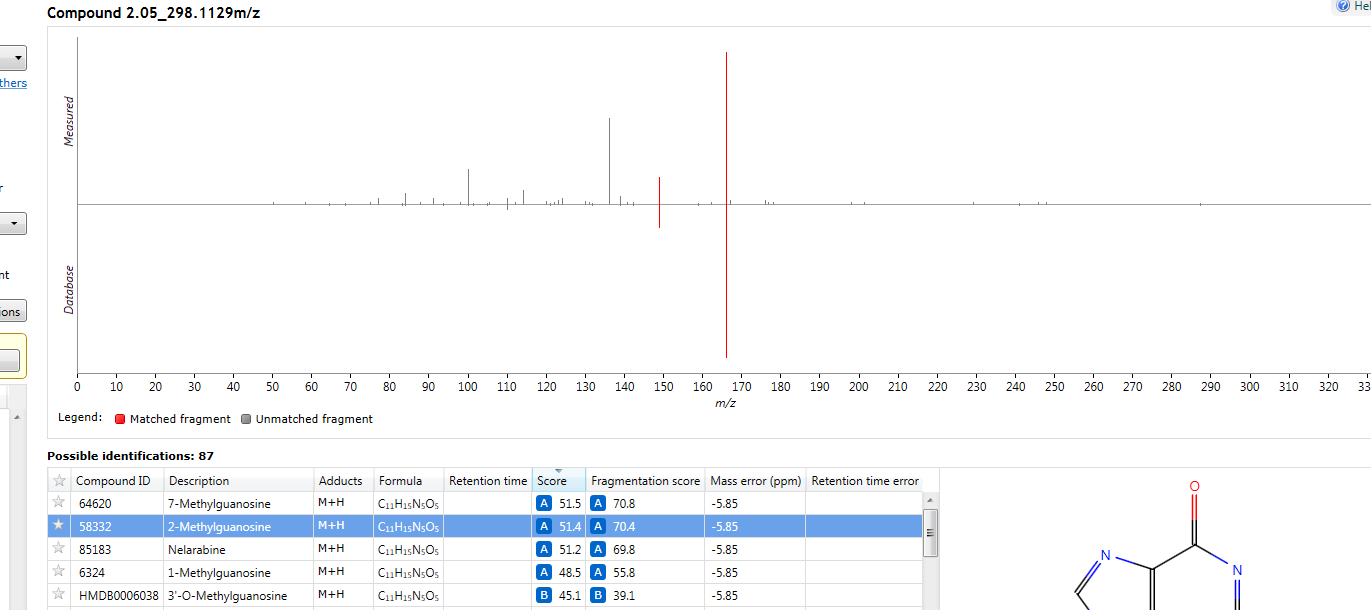
**

**5-Hydroxy-L-tryptophan (Score:50.6; Fragmentation score:71.8)**

**
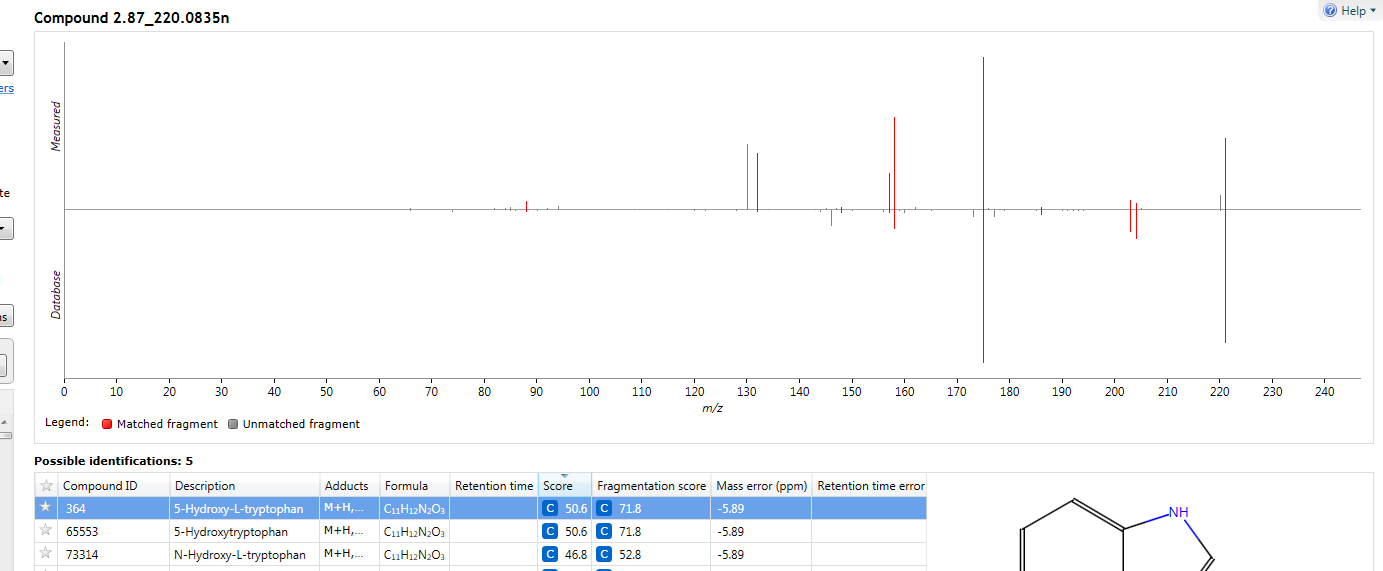
**

**7-Aminomethyl-7-carbaguanine (Score:47.5; Fragmentation score:51.7)**

**
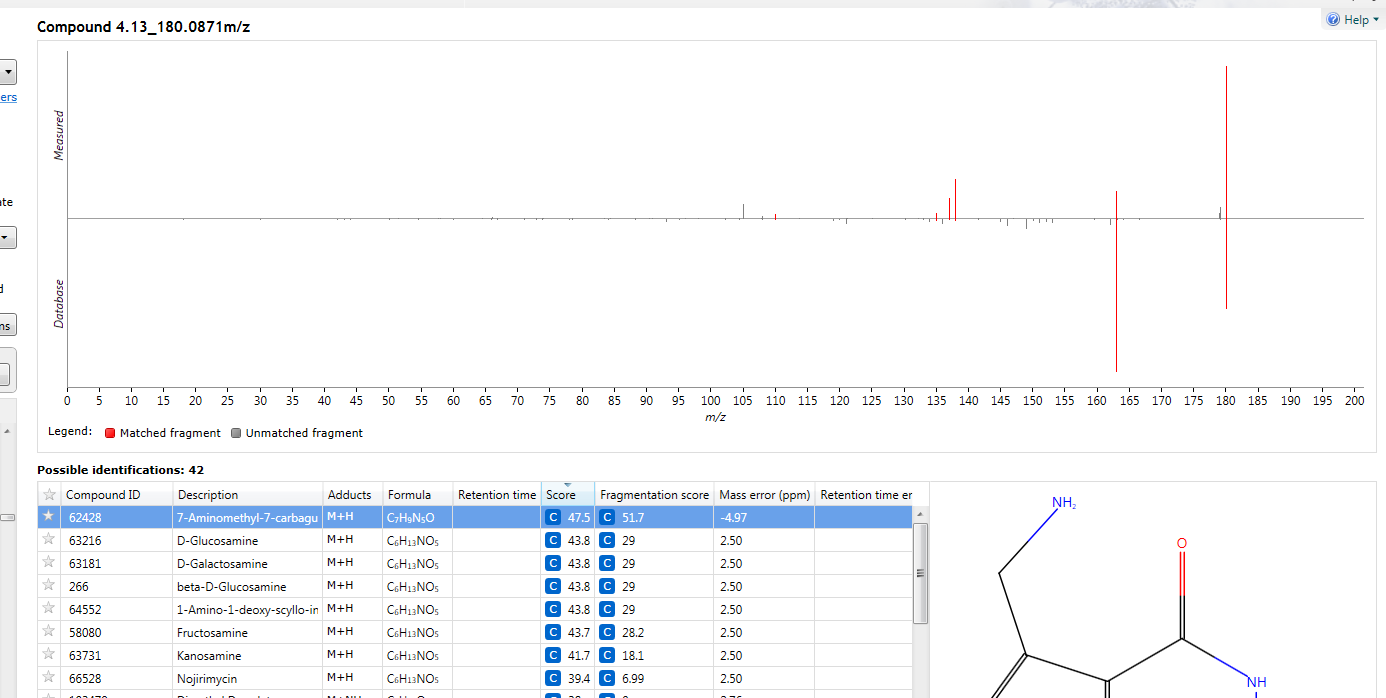
**

**Androstenol (Score:51.3; Fragmentation score:68)**

**
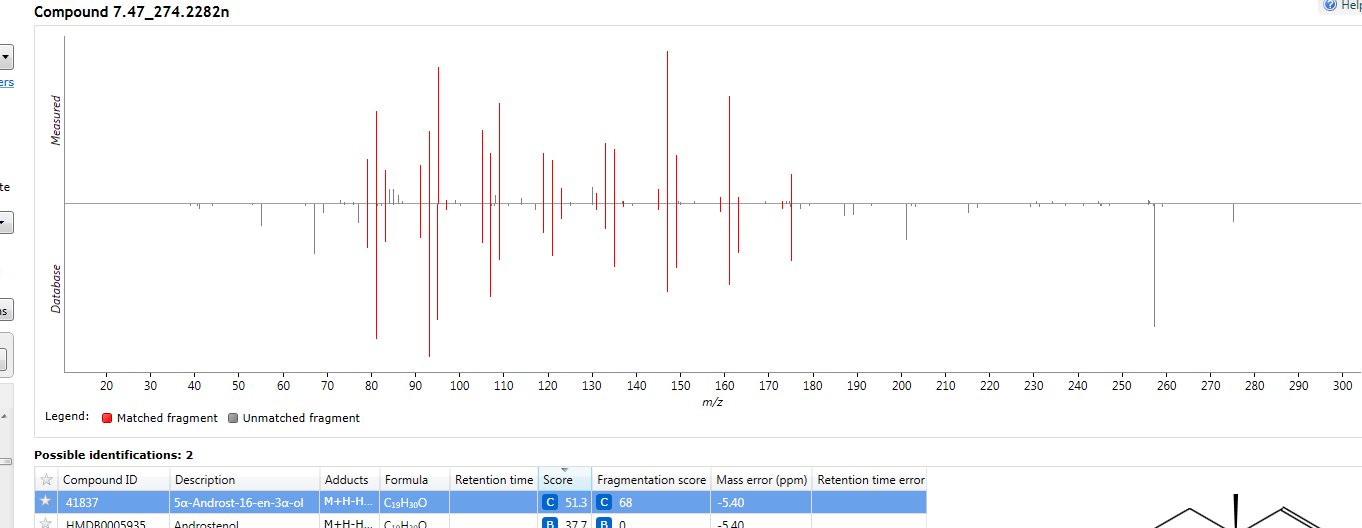
**

**Butyrylcarnitine (Score:47.9; Fragmentation score:58.6)**

**
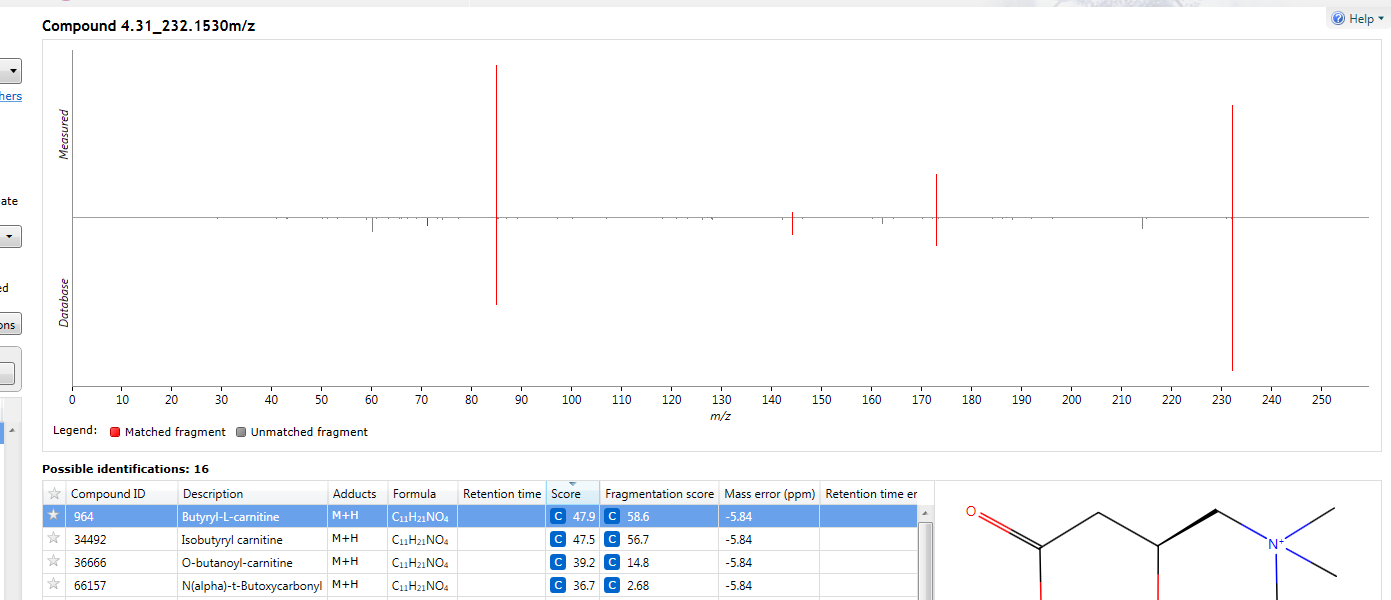
**

**Isonicotinylglycine (Score:48; Fragmentation score:55.1)**

**
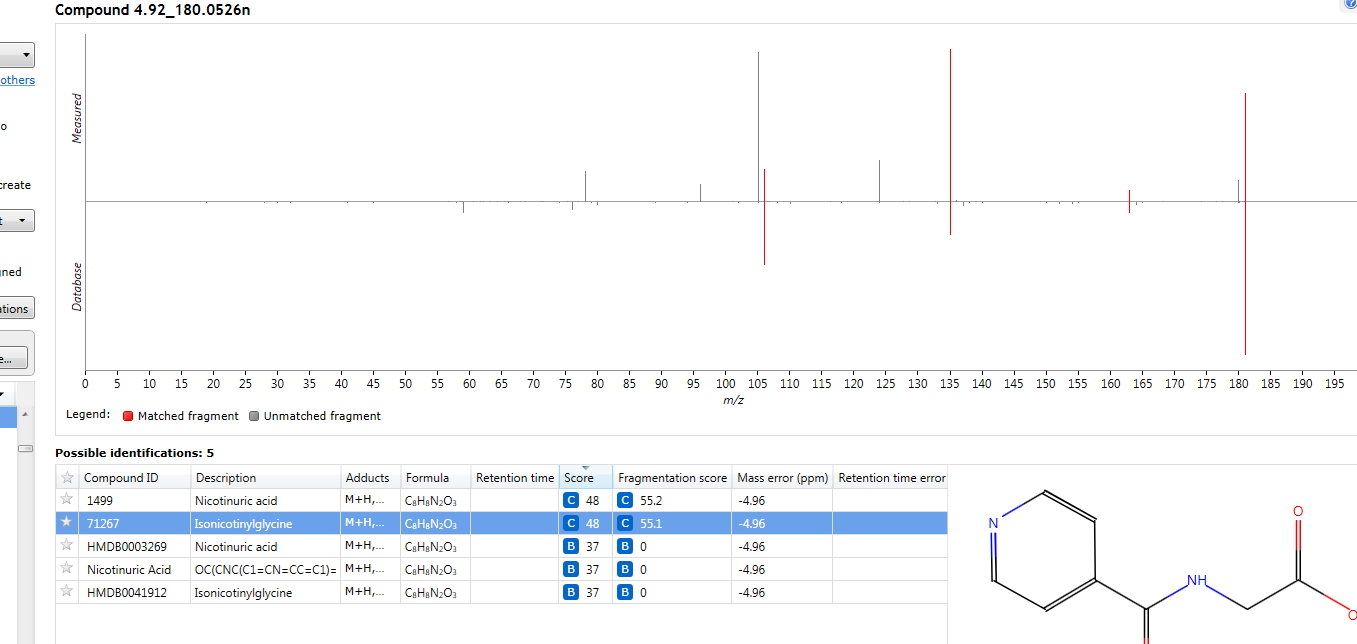
**

**N2,N2-Dimethylguanosine (Score:53.2; Fragmentation score:74.7)**

**
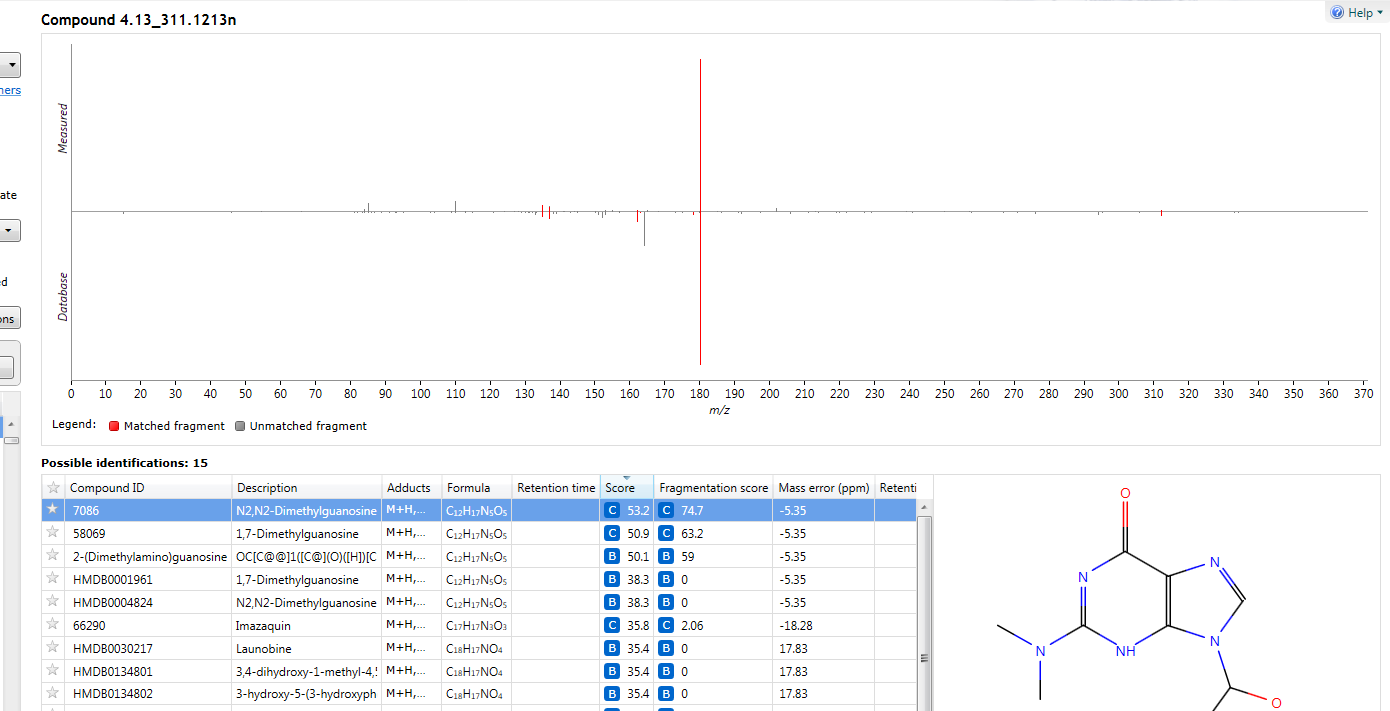
**

**Cytosine (Score:43.7; Fragmentation score:29.4)**

**
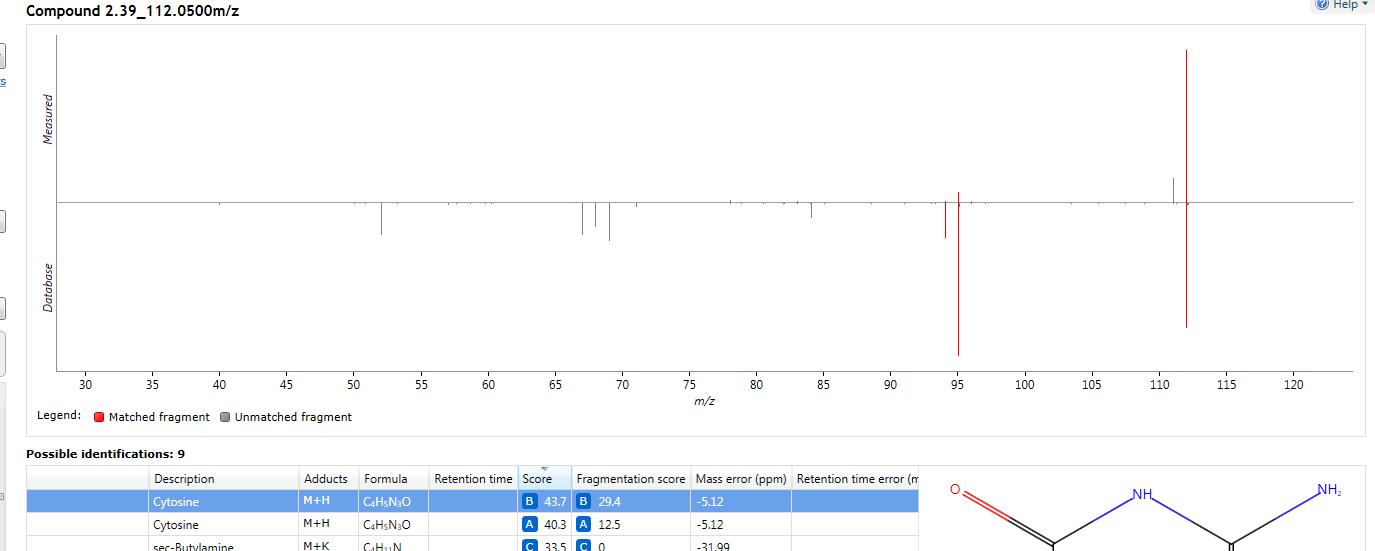
**

**N-Acetylaspartylglutamic acid (Score:47.4; Fragmentation score:48)**

**
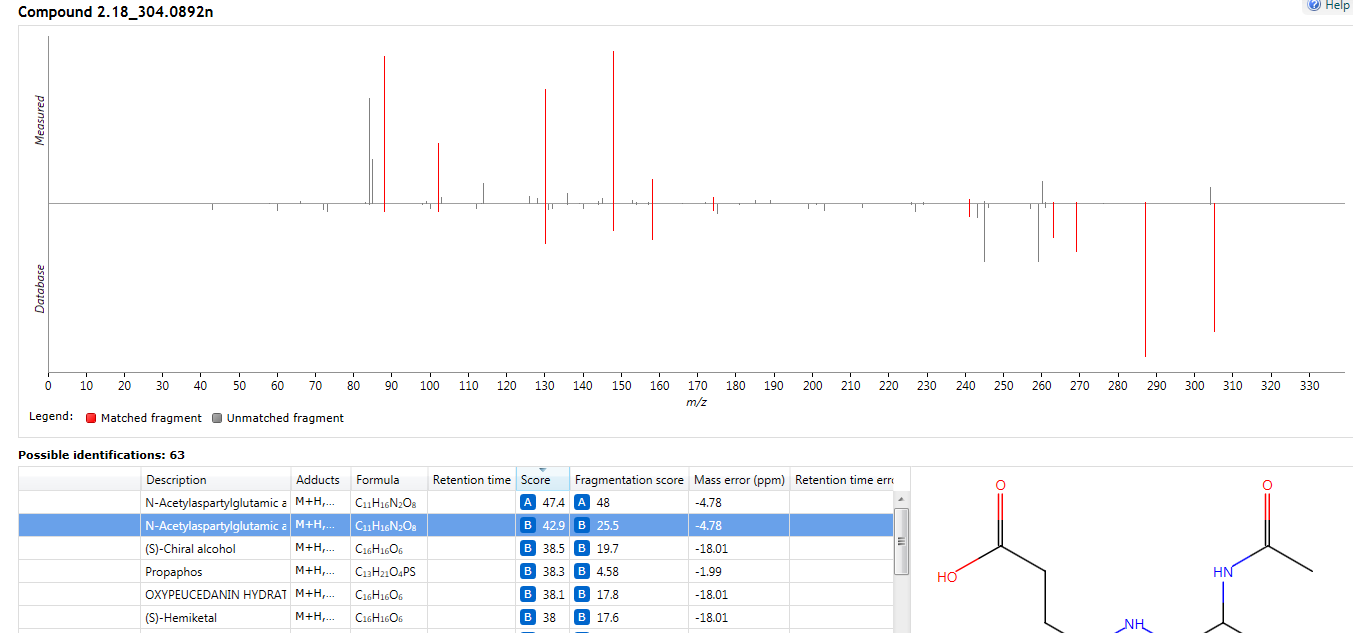
**

**Isoleucyl-Hydroxyproline (Score:39.6; Fragmentation score:14.7)**

**
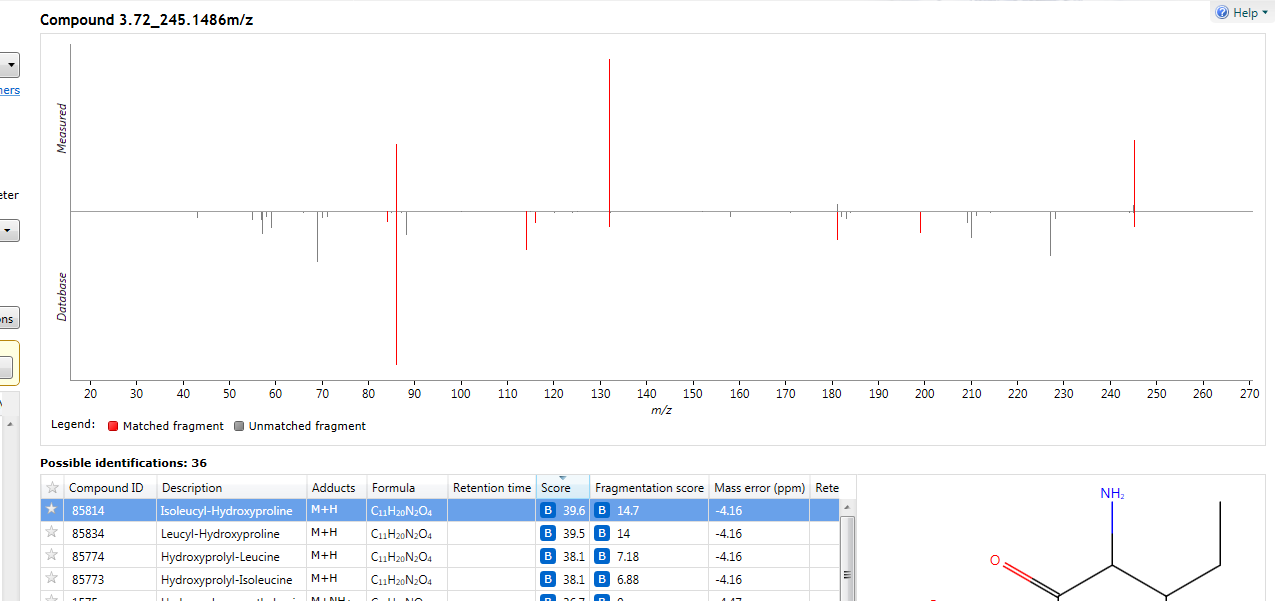
**

**3-Methylxanthine (Score:45.6; Fragmentation score:40.1)**

**
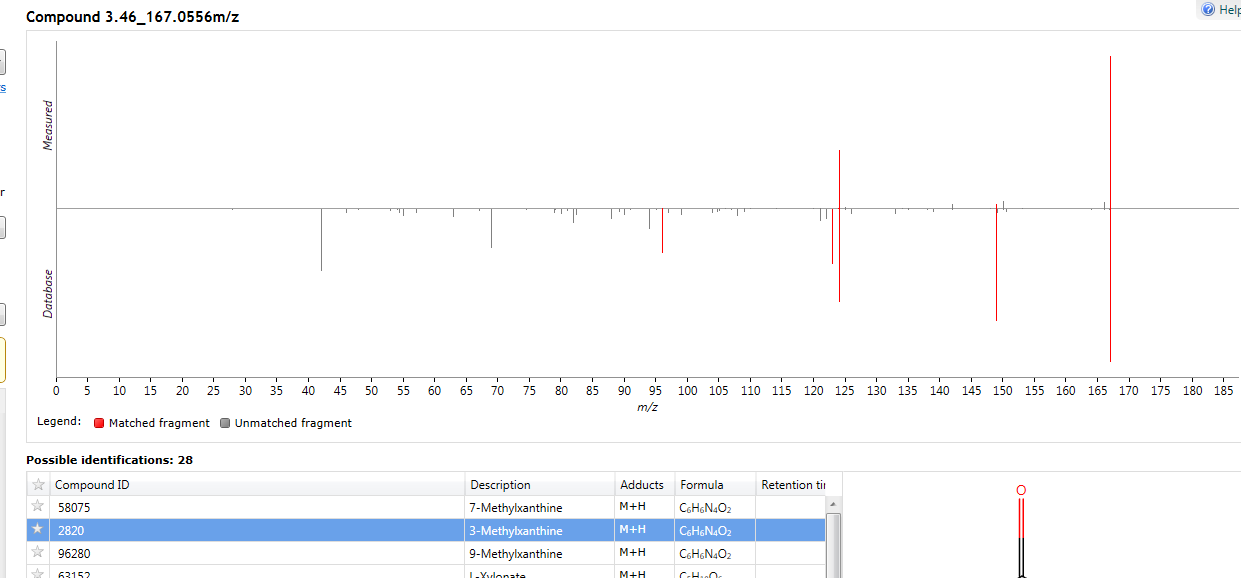
**

**L-phenylalanyl-L-hydroxyproline (Score:45.3; Fragmentation score:43.2)**

**
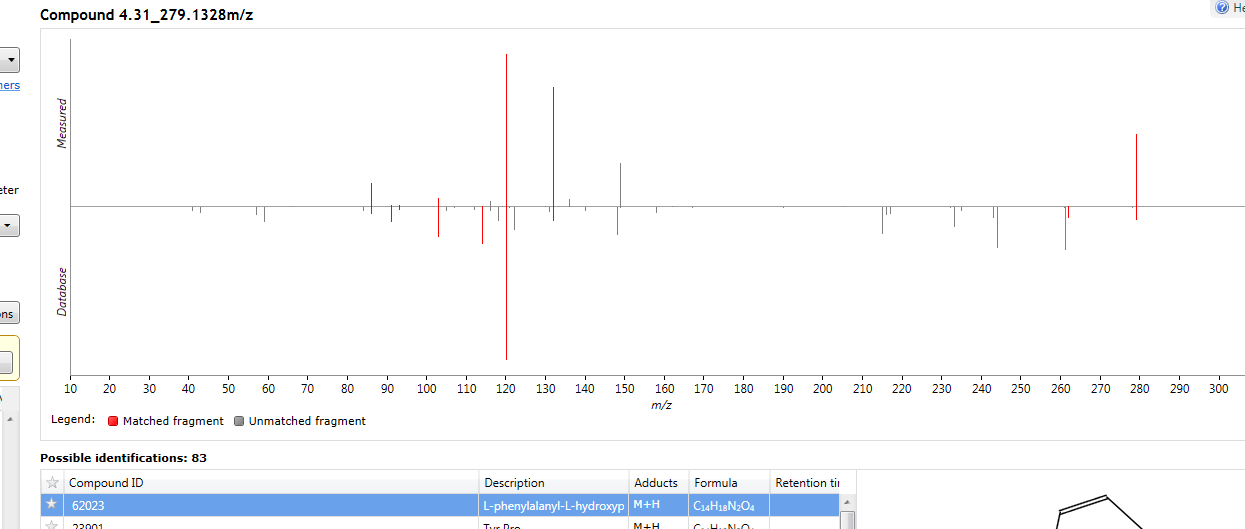
**

**4-(2-Aminophenyl)-2,4-dioxobutanoic acid (Score:47; Fragmentation score:54.2)**

**
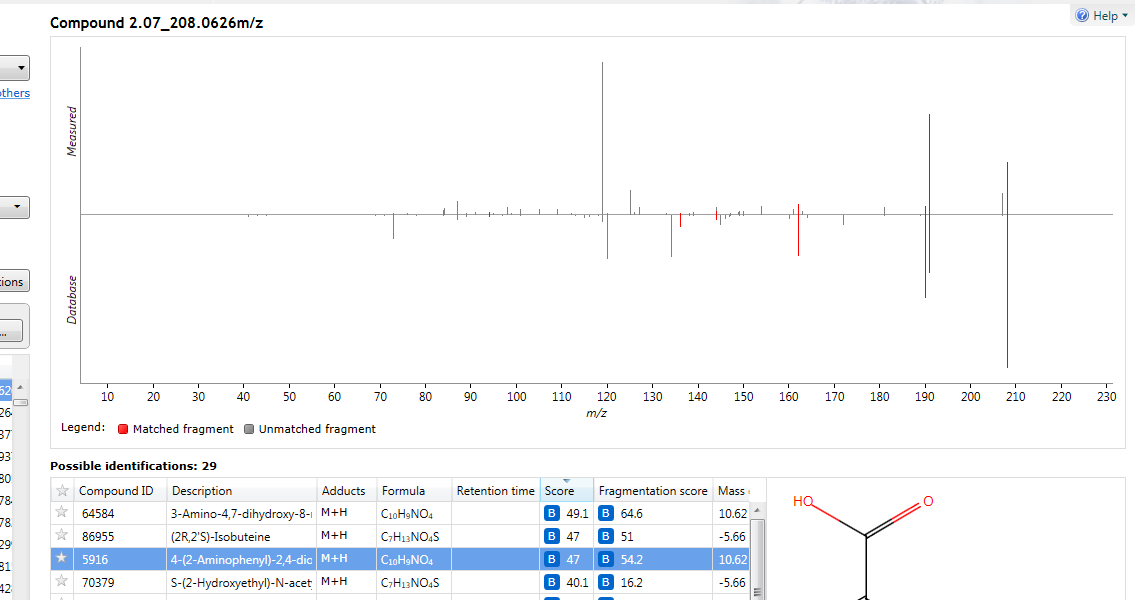
**

**Guanidoacetic acid (Score:49; Fragmentation score:54.3)**

**
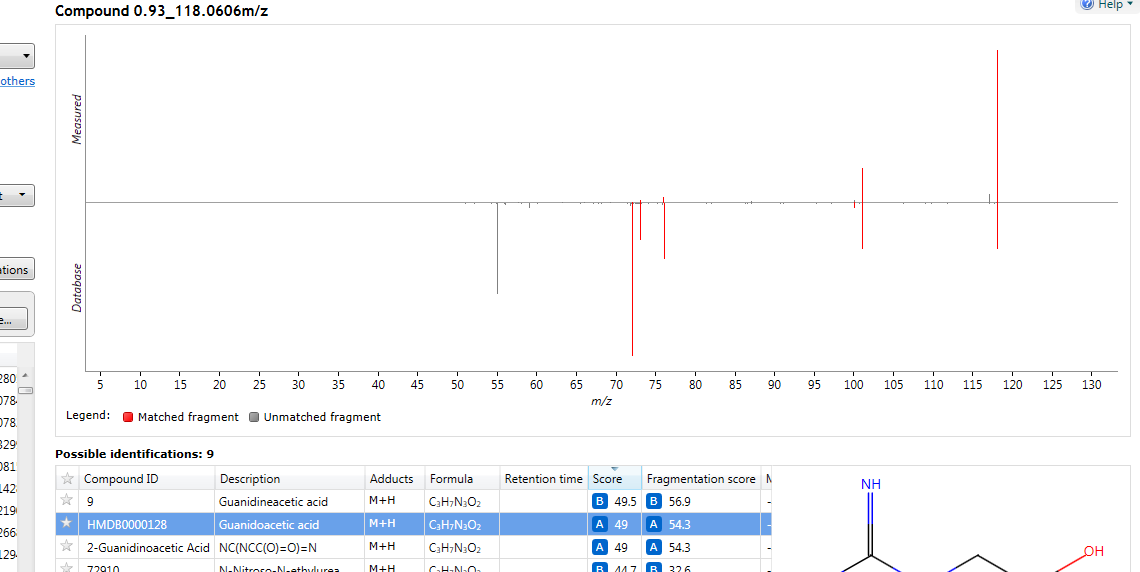
**

**L-Formylkynurenine (Score:50; Fragmentation score:60.7)**

**
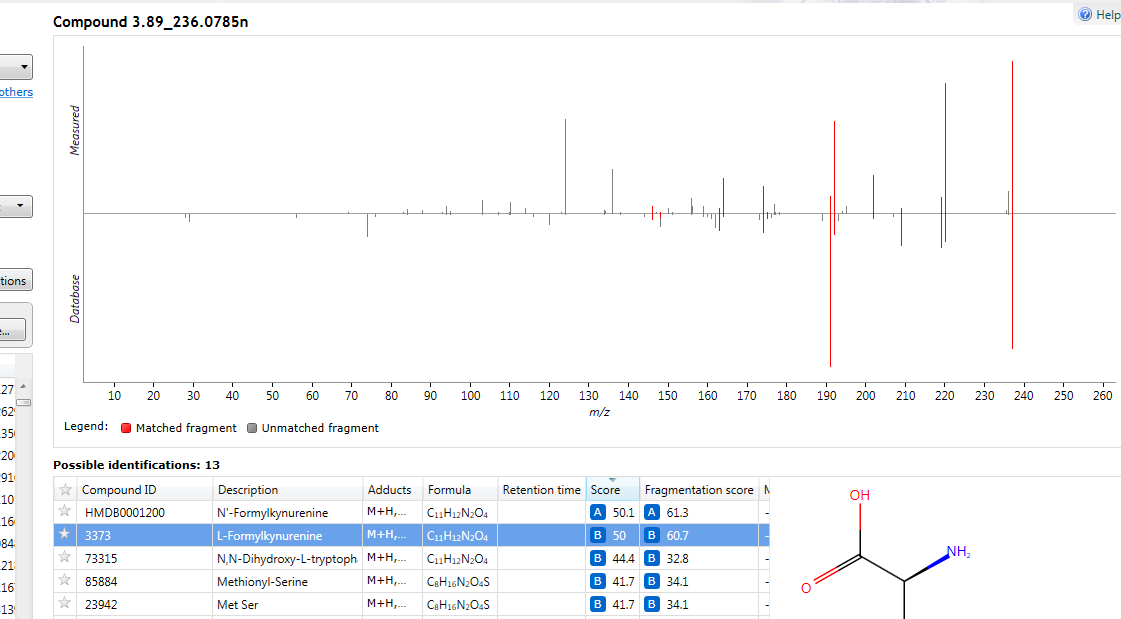
**

**N-acetyltryptophan (Score:46.3; Fragmentation score:54.7)**

**
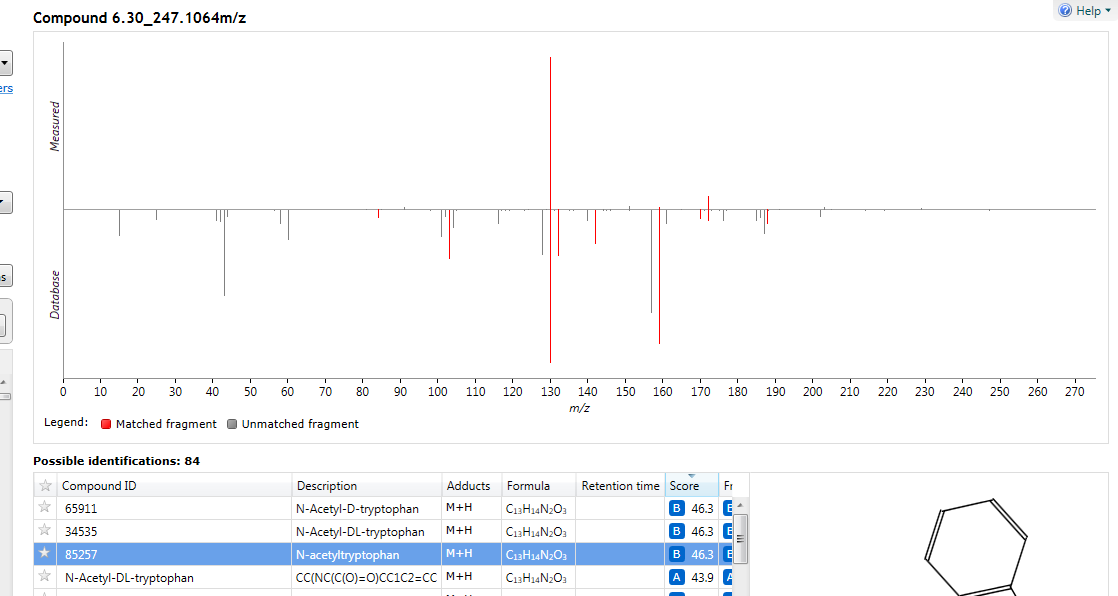
**

**L-beta-aspartyl-L-phenylalanine (Score:46.4; Fragmentation score:42.6)**

**
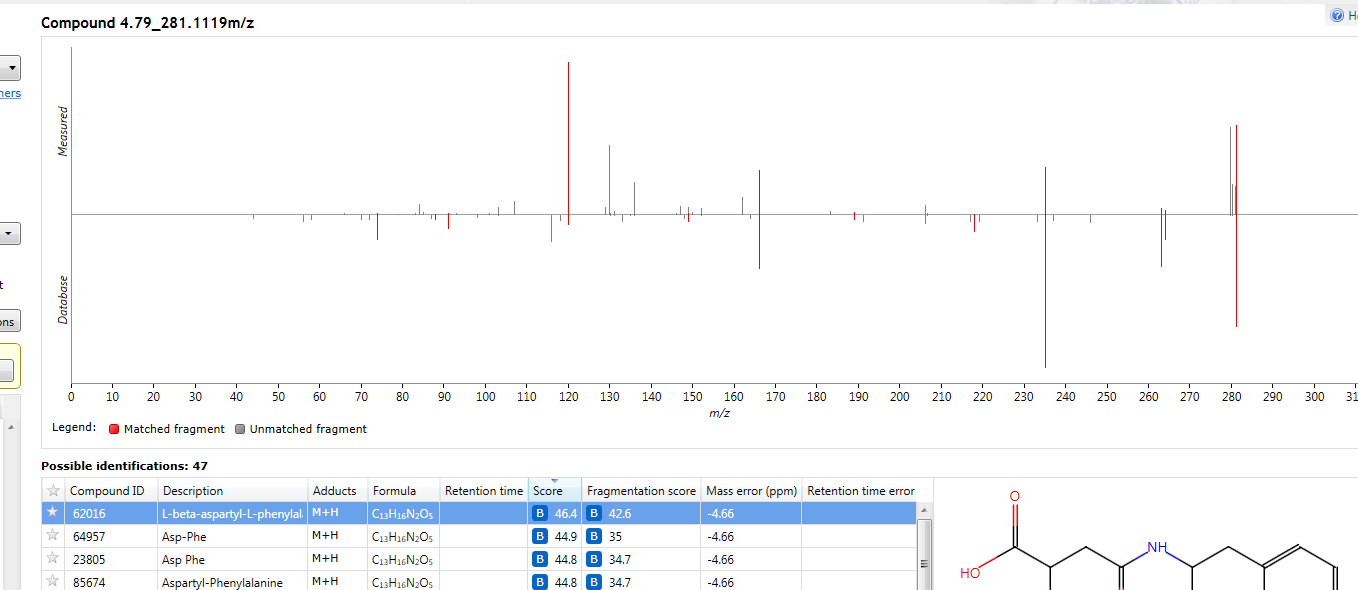
**

**8-Hydroxy-7-methylguanine (Score:45.7; Fragmentation score:41.9)**

**
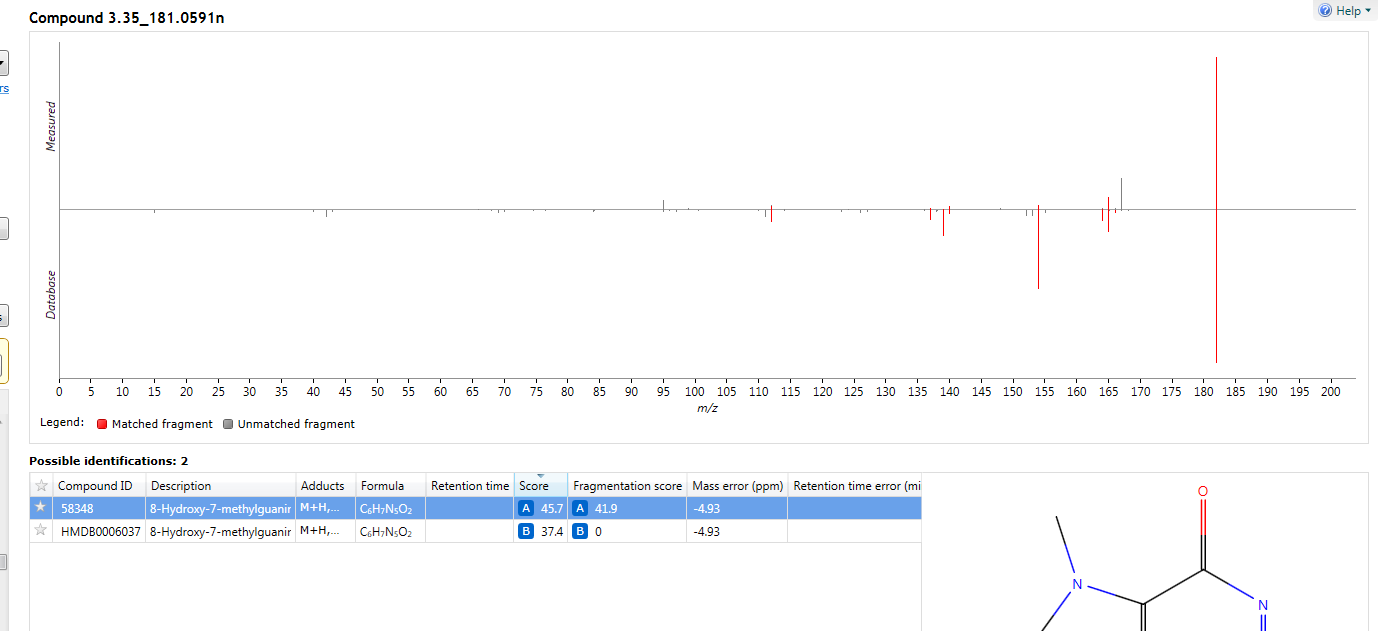
**

**11-beta-Hydroxyandrosterone-3-glucuronide (Score:46.7; Fragmentation score:39.8)**

**
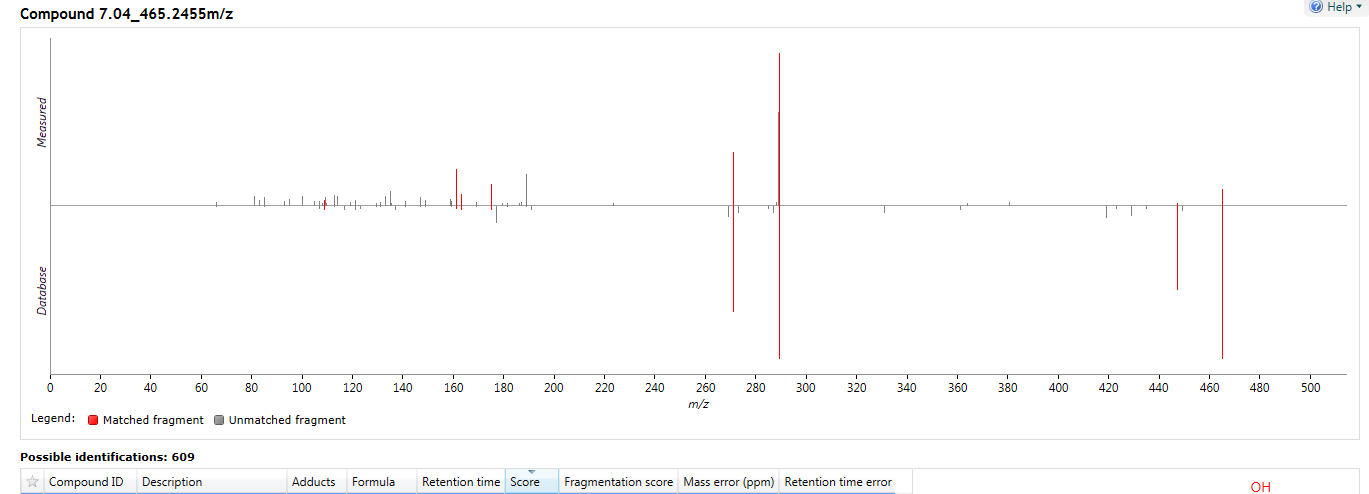
**

**Guanine (Score:45.2; Fragmentation score:41.4)**

**
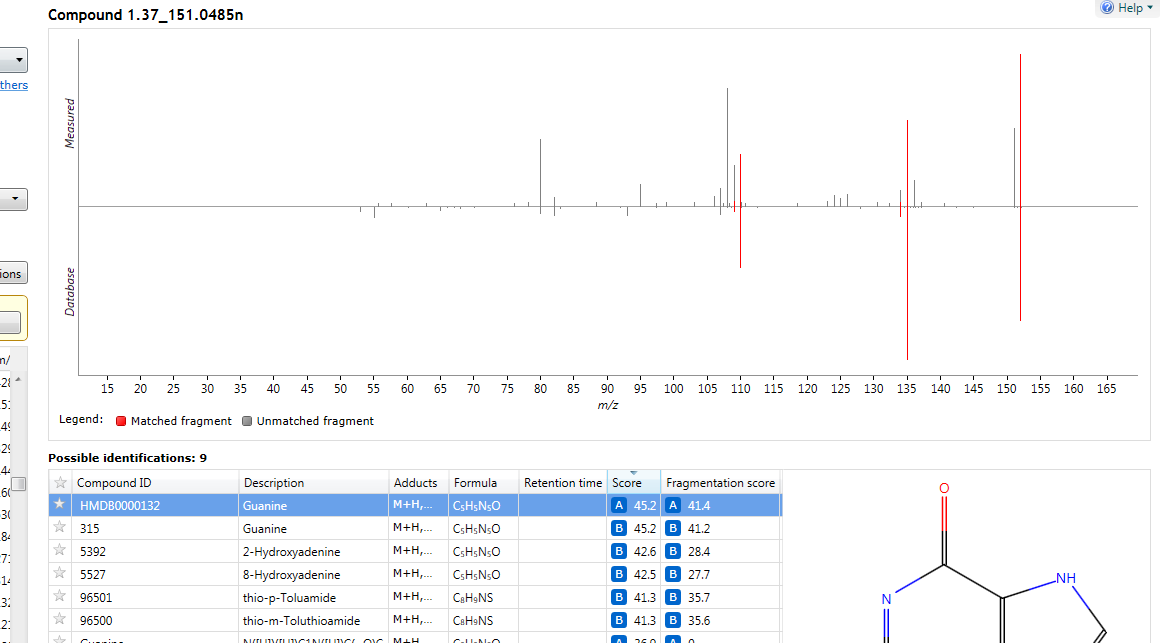
**

**Oxypurinol (Score:48.8; Fragmentation score:57.5)**

**
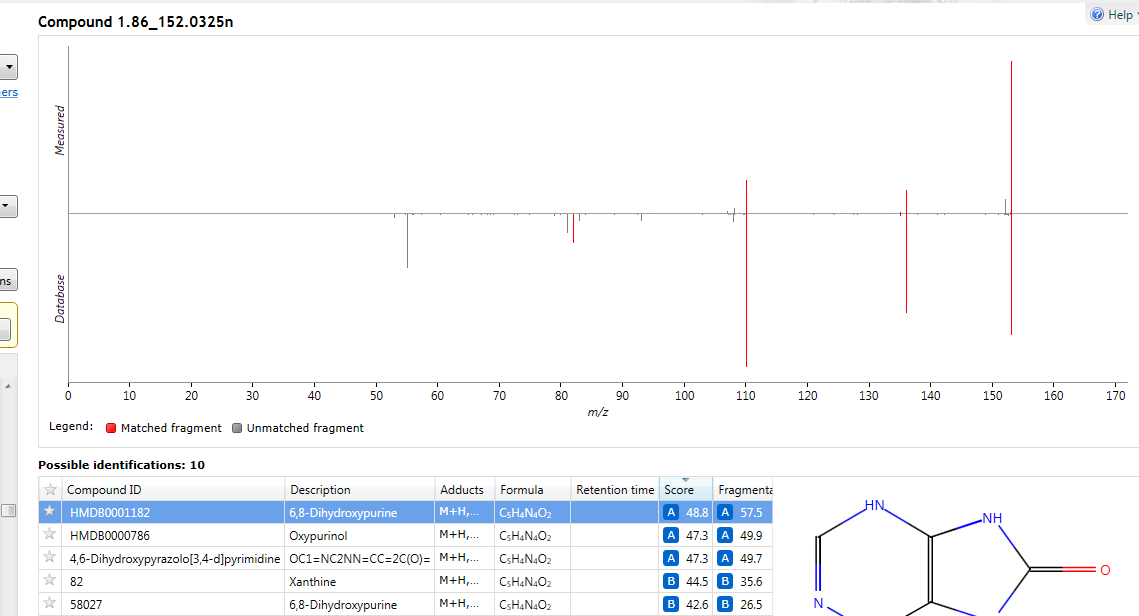
**

**Testosterone glucuronide (Score:48.2; Fragmentation score:54.9)**

**
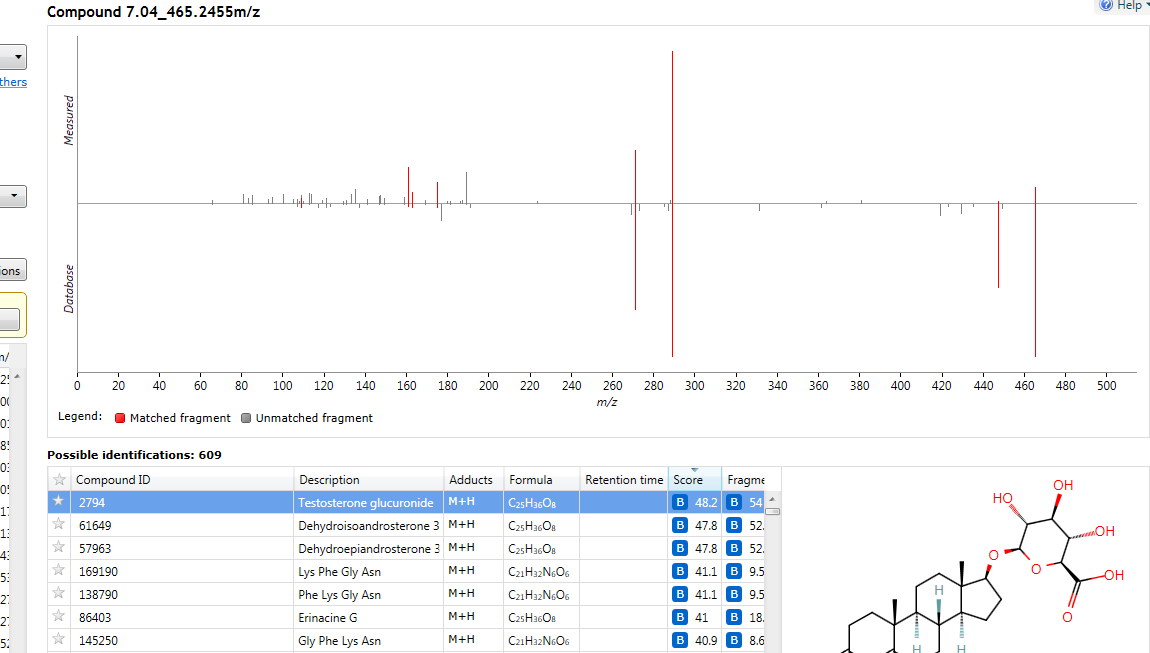
**

**L-prolyl-L-proline (Score:47; Fragmentation score:45.6)**

**
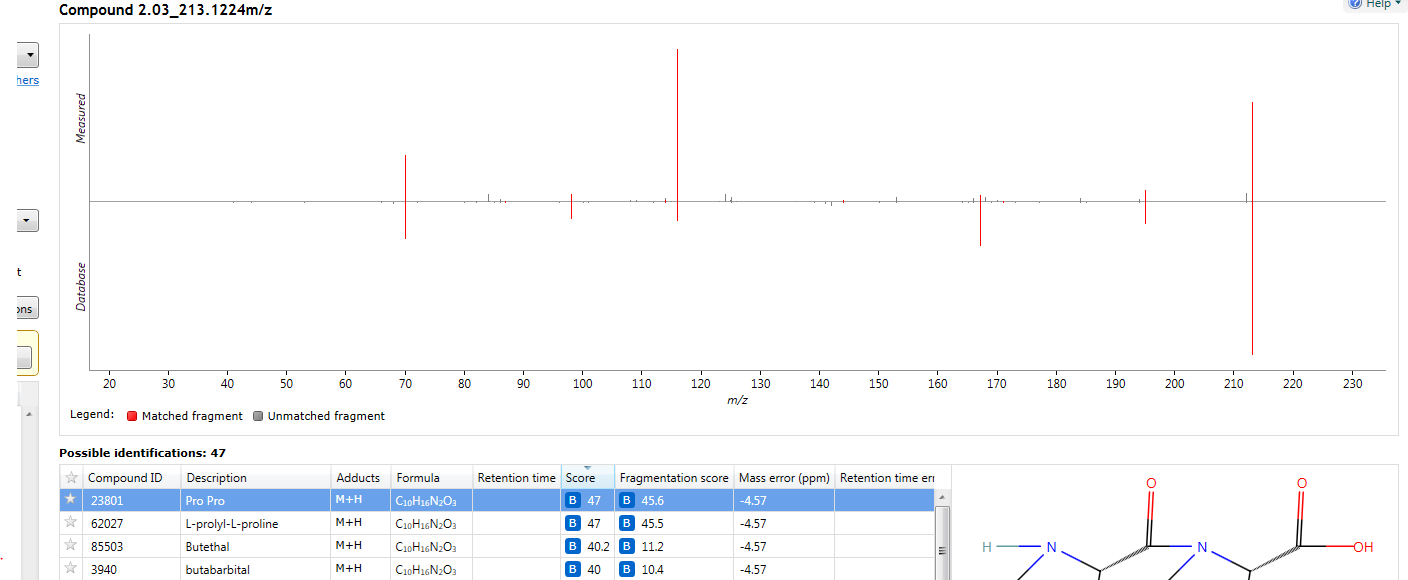
**

**L-Valine (Score:42.2; Fragmentation score:21.4)**

**
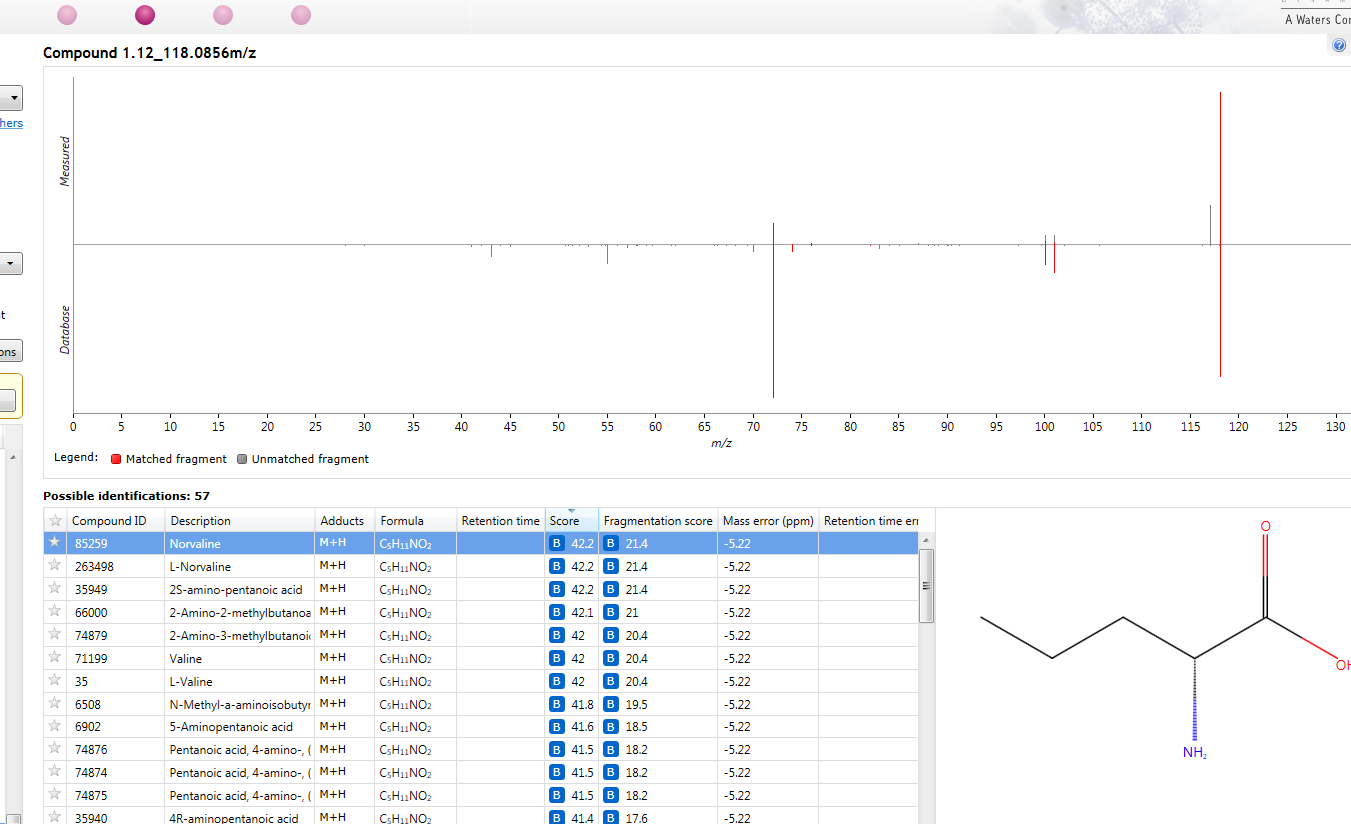
**

**Cotinine (Score:45.8; Fragmentation score:46.2)**

**
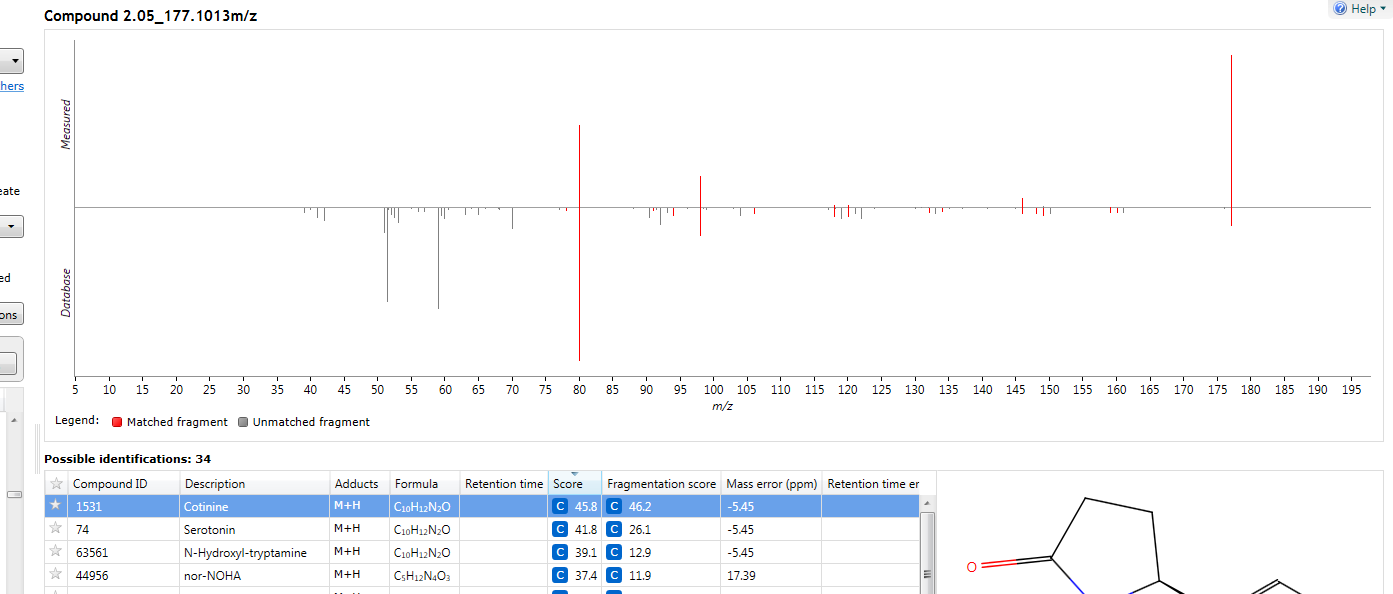
**

**1-Methylhistidine (Score:48.6; Fragmentation score:54.9)**

**
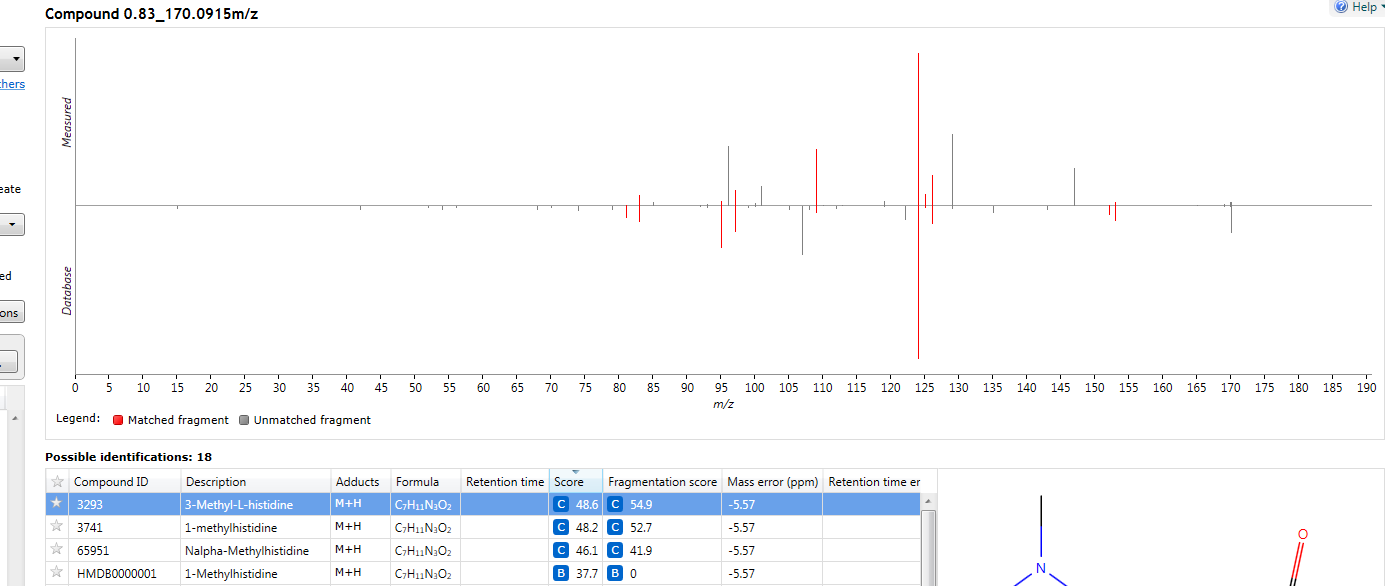
**

**Asparaginyl-Valine (Score:45.1; Fragmentation score:43.8)**

**
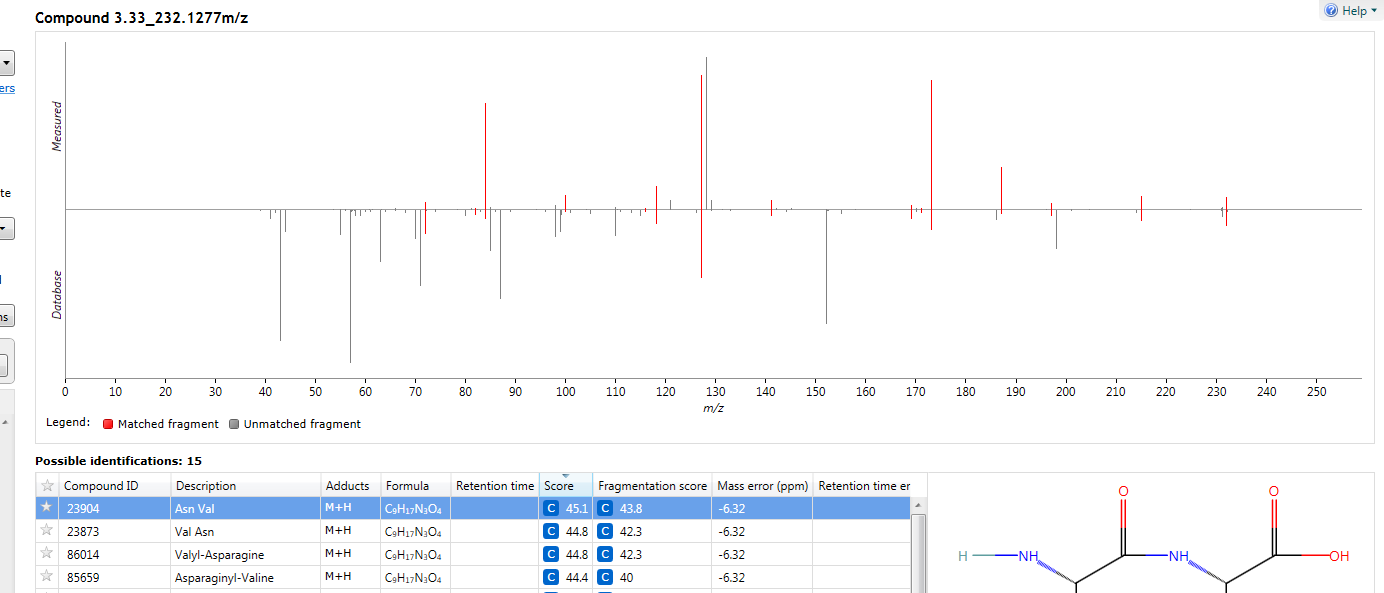
**

**Pyroglutamic acid (Score:51.8; Fragmentation score:70)**

**
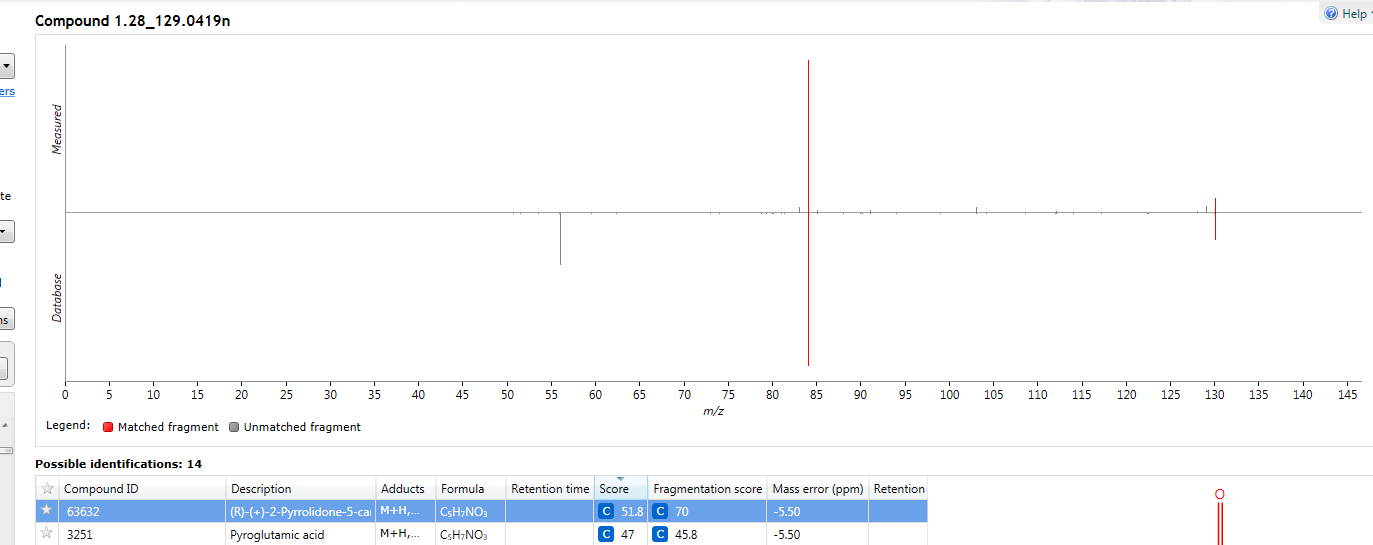
**

**Threoninyl-Proline (Score:46.6; Fragmentation score:50.6)**

**
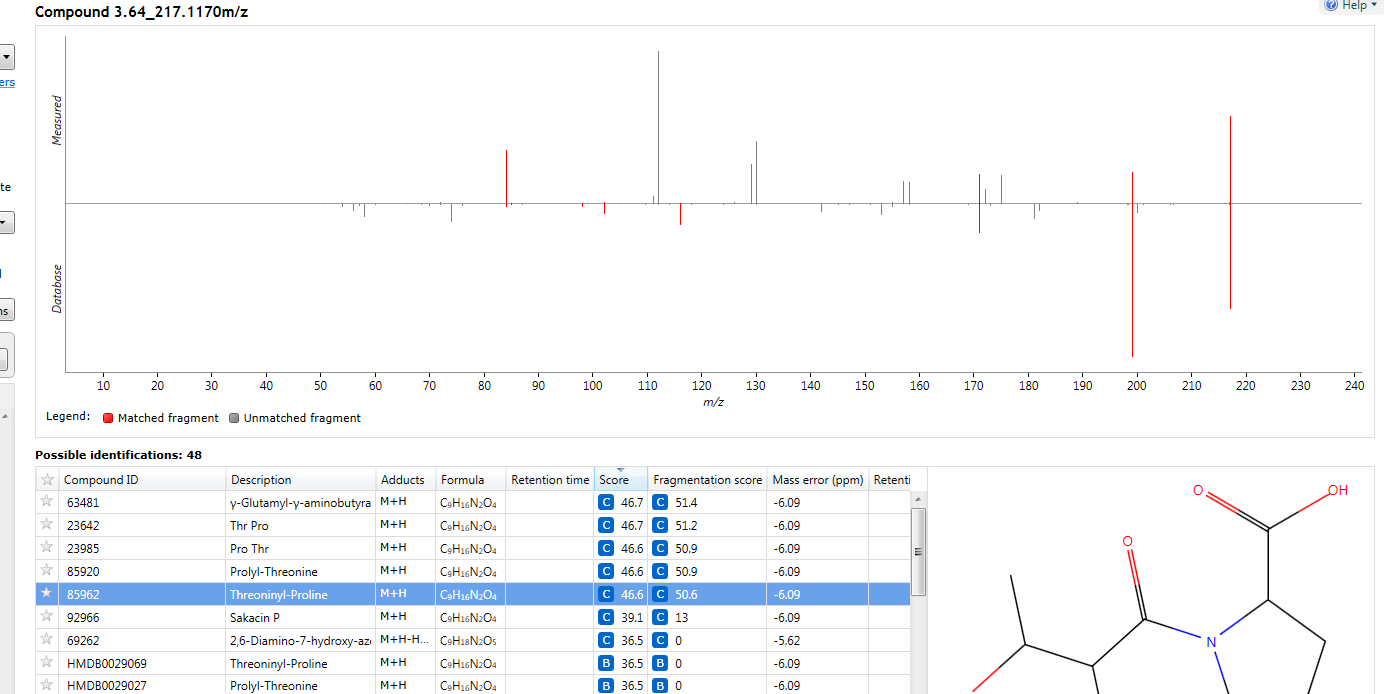
**

Fig S4 Metabolites variation with age in females (girls).


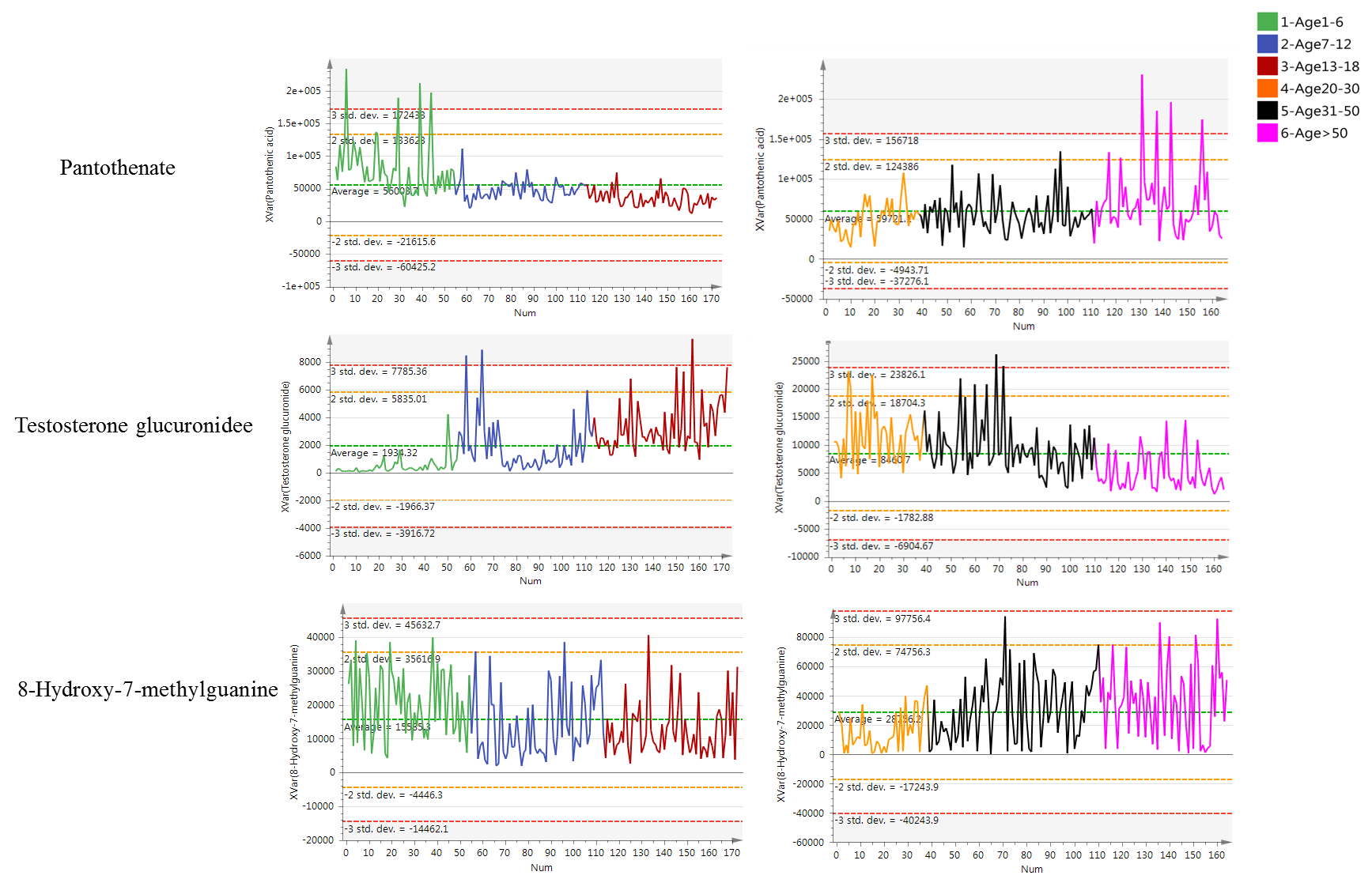

Supplement: Supplemental Information 2 [file peerj-10-13545-s002.docx]
